# The Response and Recovery of Carbon and Water Fluxes in Australian Ecosystems Exposed to Severe Drought

**DOI:** 10.1111/gcb.70361

**Published:** 2025-07-25

**Authors:** C. Stephens, B. Medlyn, L. Williams, J. Knauer, A. Inbar, E. Pendall, S. K. Arndt, J. Beringer, C. M. Ewenz, N. Hinko‐Najera, L. B. Hutley, P. Isaac, M. Liddell, W. Meyer, C. E. Moore, J. Cranko Page, R. Silberstein, W. Woodgate

**Affiliations:** ^1^ Hawkesbury Institute for the Environment Western Sydney University Richmond New South Wales Australia; ^2^ School of Agriculture, Food and Ecosystem Sciences The University of Melbourne Parkville Victoria Australia; ^3^ School of Agriculture and Environment The University of Western Australia Crawley Western Australia Australia; ^4^ Centre for Water and Spatial Science The University of Western Australia Crawley Western Australia Australia; ^5^ TERN Ecosystem Processes Central Node Airborne Research Australia Parafield South Australia Australia; ^6^ Faculty of Science and Technology Charles Darwin University Darwin Northwest Territories Australia; ^7^ OzFlux Central Node, TERN‐OzFlux Melbourne Victoria Australia; ^8^ Centre for Tropical Environmental and Sustainability Science, College of Science and Engineering James Cook University Cairns Queensland USA; ^9^ School of Biological Sciences The University of Adelaide Adelaide South Australia Australia; ^10^ Climate Change Research Centre University of New South Wales Sydney New South Wales Australia; ^11^ ARC Centre of Excellence for Climate Extremes Sydney New South Wales Australia; ^12^ School of Science Edith Cowan University Joondalup Western Australia Australia; ^13^ School of the Environment The University of Queensland St Lucia Western Australia Australia; ^14^ CSIRO, Space and Astronomy Kensington Western Australia Australia

**Keywords:** carbon fluxes, drought, ecosystems, eddy covariance, evapotranspiration, resilience, resistance

## Abstract

Climate change‐driven increases in drought risk pose a critical threat to global carbon and water cycles. However, ecosystem‐scale responses remain poorly quantified, particularly for severe, multiyear drought events. We addressed this gap by examining ecosystem‐scale carbon and water flux sensitivity to the extreme 2018–19 drought in Australia using data from 14 eddy covariance flux sites. The ecosystems span grasslands and semi‐arid woodlands to tropical and temperate forests. The driest sites (classed as “grass” and “very dry”) experienced drastic productivity impacts, with a 65% decrease in Gross Primary Productivity (GPP) over 2 years relative to the pre‐drought average. However, fluxes in “dry,” “seasonally wet” and “wet” ecosystems showed remarkable resistance, with no overall change in GPP. All sites recovered rapidly; carbon fluxes in the first post‐drought year matched (and generally exceeded) those of a climatically similar pre‐drought year. Drought responses were strongly mediated by ecosystem‐specific strategies. The driest ecosystems showed direct coupling of productivity to water availability, while intermediate ecosystems (dry and seasonally wet) leveraged stored soil water to maintain evapotranspiration and productivity under drought. At these sites, water was conserved over wet periods (evapotranspiration < demand, despite sufficient rainfall) and consumed over dry periods (evapotranspiration > rainfall). This mechanism mitigating periodic water stress under high rainfall variability likely contributed to the notable drought resistance of the dry and seasonally wet sites. The monthly water deficit index (MWDI) emerged as a robust predictor of productivity across space, highlighting that short‐term water availability deficits strongly influence overall ecosystem composition. Analysis of drought response mechanisms suggested rapid leaf loss under water stress, particularly at the driest sites. Our findings underscore the importance of accounting for sub‐surface water storage and diverse drought response strategies in vegetation models. We provide critical benchmarks for improving parameterization of plant‐water relations, aiding efforts to inform climate‐robust management strategies.

## Graphical abstract

We examined flux data across a range of Australian ecosystems exposed to severe drought over two years (2018‐19). The driest ecosystems experienced large productivity declines, but intermediate ecosystems (still generally considered water‐limited) showed no overall change in productivity, indicating surprising drought resistance. We present evidence that overall productivity in these ecosystems is limited by periodic exposure to water stress, and the associated balance between water consumption and storage enables high drought resistance.

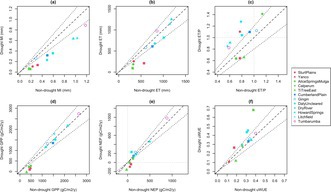

## 1 Introduction

Severe drought can damage the health of ecosystems and reduce their capacity to store carbon (Wolf and Paul‐Limoges 2023). Increasing water stress is projected to have a detrimental impact on vegetation productivity under rising temperatures (Seneviratne et al. 2023), and this is often cited as a key threat to the future global carbon balance (Sharma et al. 2023; Uribe et al. 2023). However, quantifying and forecasting drought response at a whole–ecosystem scale remains a challenge due to limited observational data, particularly from severe, multiyear droughts.

Models of vegetation response to drought are typically parameterized with data measured at the leaf or whole‐plant scale, and there are significant uncertainties in how these responses translate to canopy, ecosystem, or larger scales. Intercomparisons among models show substantial disagreement in simulated drought sensitivities for a given vegetation type (Medlyn et al. 2016), while data‐model comparisons disagree on both the magnitude and sign of errors in modeled drought sensitivity. Bastos et al. (2020) compared GPP sensitivity to summer drought in 11 vegetation models to that of the FLUXCOM product, which is based on flux tower observations, and found models largely underestimated the FLUXCOM drought sensitivity. This result aligns with other studies indicating that models underestimate the sensitivity of canopy conductance to decreasing soil water availability as inferred from land surface temperatures (Green et al. 2024), and that models underestimate drought effects on productivity, as inferred from flux measurements (MacBean et al. 2021) and tree‐ring data (Kolus et al. 2019). In contrast, Fu et al. (2024) showed that models often overestimate the soil water threshold at which evapotranspiration (ET) becomes water‐limited (particularly in dry climates), which could lead them to overestimate drought sensitivity. Other studies have also shown overly large declines in simulated ET compared to observations under low‐rainfall conditions (Giardina et al. 2023; Ukkola et al. 2016). To develop more data‐informed model parameterizations, there is a clear need for deeper quantitative understanding of ecosystem‐scale carbon and water flux responses to severe drought.

One important question is how much carbon and water flux responses to drought vary across ecosystem types. It is typically observed that carbon fluxes in dry ecosystems show stronger water sensitivity (i.e., lower drought resistance) than those in wet ecosystems (e.g., Beringer et al. (2022), Haverd et al. (2017)). Huxman et al. (2004) analyzed 14 American sites across a precipitation (*P*) gradient and found that the response of productivity to *P* was steepest at the driest sites. Biederman et al. (2016) similarly found a saturating relationship between *P* and GPP across a sample of relatively dry sites, with steeper response slopes at drier sites. Giardina et al. (2023) derived a water stress factor based on the ratio of observed ET to potential ET (PET) and found larger declines under reduced water availability at grasslands and savannas than forests. These results suggest a higher ability to maintain ET in forests (at least under mild drought), consistent with other research showing relatively low drought sensitivity in forests (Deng et al. 2023; Hoek van Dijke et al. 2023). Over a 4‐year drought in the western United States, Schwalm et al. (2012) showed larger ET decreases in forests than grasslands or savannas, while grasslands had the largest GPP declines. During a shorter (4‐month) but very hot drought in the same region, Dannenberg et al. (2022) calculated large GPP reductions across a range of ecosystem types, highlighting the role of heat stress and evaporative demand in drought response. Drought‐induced reductions in ecosystem respiration (ER) could lessen the impact of water stress on carbon exchange, but high temperatures may increase ER and hence negate this compensatory effect (von Buttlar et al. 2018).

A second critical question is the length of time taken for recovery after a drought breaks (Schwalm et al. 2017). Recovery dynamics can be complicated by legacy effects on vegetation (e.g., xylem damage, mortality (Nolan et al. 2021)) and/or impacts of drought‐related disturbances (e.g., fires associated with dry biomass (Boer et al. 2020)), as well as nuances of post‐drought climate (Stephens et al. 2023). Zhang et al. (2021) used eddy covariance data to investigate recovery time for gross primary productivity (GPP) after drought and found that arid ecosystems (which are particularly well adapted to “bounce back” after drought) and humid ecosystems (which may not be water‐limited even under drought) recovered more quickly than either sub‐humid or semi‐arid ecosystems. They also noted faster recovery times (i.e., higher drought resilience) in grasslands and shrublands than forests. Over two consecutive summer droughts in Europe, Bastos et al. (2021) showed greater‐than‐expected reductions in greenness during the second drought, presumably associated with legacy effects of the first.

Understanding and modeling ecosystem drought response must consider the different mechanisms by which plants manage water limitation and avoid xylem embolism (Blackman et al. 2019; Nardini and Salleo 2000). Common responses to water stress include stomatal closure and/or leaf abscission to reduce transpiration, concurrently downregulating photosynthesis (Bucci et al. 2005; Eamus et al. 2008). While these strategies reduce a plant's rate of water loss, they can compromise productivity over time (Blackman et al. 2019). Non‐stomatal limitations on photosynthesis under stress (e.g., reduced electron transport efficiency, reduced mesophyll conductance) may also contribute to loss of ecosystem productivity (Gourlez de la Motte et al. 2020; Nelson et al. 2018; Yang et al. 2019).

Questions around vegetation response to severe, multiyear droughts have been difficult to address due to a lack of observational data capturing such events at a wide enough scale to encompass a variety of ecosystem types. In 2018–19, Australia experienced extreme drought across most of the continent (Devanand et al. 2024; Fang et al. 2021; King et al. 2020), with 2019 reported as the hottest and driest year across *a* > 100‐year record (Bureau of Meteorology 2020). These conditions were attributed to a strong El Niño coupled with positive Indian Ocean Dipole and negative Southern Annular Mode events (Nguyen et al. 2021). These drivers of climatic variability were superimposed on an overall warming trend (Bureau of Meteorology and CSIRO 2024) along with decreasing relative humidity (Denson et al. 2021) and rising evaporative demand (Stephens et al. 2018). Extensive drought‐induced dieback was observed (Losso et al. 2022; Nolan et al. 2021; Wright et al. 2023) and continued dry conditions led to unprecedented fire in the summer of 2019/20 (Boer et al. 2020). High quality flux data are available over many affected sites before, during, and after the 2018–19 drought through the TERN‐OzFlux eddy covariance site network (Beringer et al. 2016, 2022), presenting a valuable opportunity to better understand drought effects on carbon and water fluxes.

Here we use these data to investigate the responses of carbon and water fluxes to severe drought at 14 contrasting sites spanning the Australian continent. The ecosystem types captured by the network range from grasslands and semi‐arid woodlands to tropical savannas and wet temperate forests. We first examine (1) how much the severe 2018–19 drought affected carbon and water fluxes across different vegetation types, and (2) was there evidence of post‐drought legacy effects on productivity? We then explore the mechanisms underpinning contrasting drought effects on ecosystem‐water relations, asking (3) can we explain the drought responses by understanding the control of *P* on ET together with site disturbance histories? Finally, we examine (4) what is the relative importance of leaf abscission versus stomatal and non‐stomatal photosynthetic response at each site? The results of (1) and (2) can be used to validate drought responses in process‐based vegetation models, while (3) and (4) can inform process parameterization for different vegetation types.

## 2 Data and Methods

### 2.1 Flux and Climate Data

Eddy covariance data for this study were obtained from the Terrestrial Ecosystem Research Network's (TERN) OzFlux flux tower network (Beringer et al. 2016). We downloaded Level 6 (quality assured and gap‐filled) data for all non‐cropping sites across Australia (TERN 2023). We then used the following criteria to select sites for our analysis:

1. Data cover the 2018–19 calendar years and at least 2 years prior (i.e., must have data from 2016 to 2019 at a minimum).
2. No major data gaps or known issues during the drought period.
3. Site not considered to be intensively managed.

**FIGURE 1:**
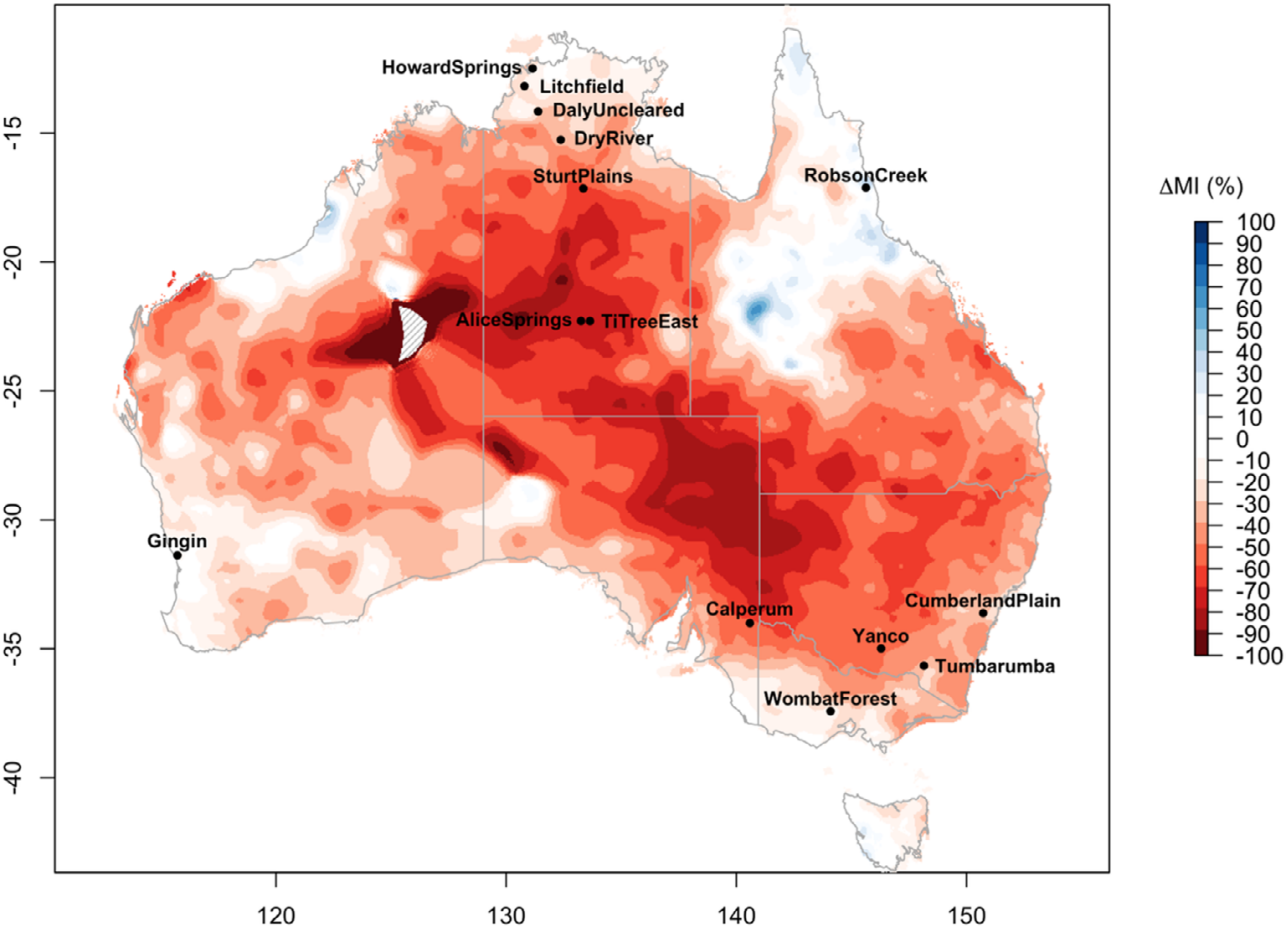
Difference in Moisture Index (MI = *P*/PET) between pre‐drought (2010–17) and drought (2018–19) periods expressed as percentage change relative to pre‐drought MI. Red areas indicate drought, with darker colours showing higher severity. Grey hatching indicates missing data.

In total, 14 sites met these requirements (3.1 Figure 1). We aimed to analyze ecosystem response to the 2018–19 drought relative to non‐drought conditions, so we excluded data prior to 2010 when some sites were affected by the Millennium Drought (van Dijk et al. 2013; van Gorsel et al. 2013). We excluded data from Tumbarumba after 2019 due to a fire on 31/12/2019, and from Calperum after June 2020 due to an instrument change after which we noted much higher NEP than in previous years. Data at Daly Uncleared was excluded from 01/11/2022 due to land clearing. The dataset provides Net Ecosystem Exchange (NEE) which is partitioned into ER and GPP using the SOLO machine learning approach (Abramowitz et al. 2006; Hsu et al. 2002). We note low confidence in ER and GPP data for the Ti Tree East site because photodegradation invalidates the assumptions required for NEE partitioning (Cleverly et al. 2016; Tarin et al. 2020). We use GPP and Net Ecosystem Productivity (NEP = ‐NEE) as indicators of productivity and flux tower ET data to indicate ecosystem water use.

Our analysis uses *P* and PET data to calculate water availability via the Moisture Index (MI = *P*/PET, see Prentice et al. (2022)). We used the gridded Australian Water Availability Project (AWAP) product to quantify rainfall (Jones et al. 2009) due to issues with OzFlux precipitation gauge accuracy and missing data. The AWAP product is an amalgamation of high‐quality climate observations across Australia, including rainfall based on quality‐controlled and appropriately sited gauges. *P* was extracted using the AWAPer package (Peterson et al. 2020) at each TERN‐OzFlux site location. A comparison between gridded rainfall and site rainfall is shown in Figure S1. Time series of PET for each site were calculated using the *Evapotranspiration* package (version 1.16) in R (Guo et al. 2016) based on the Penman equation (Penman 1948) with site‐recorded humidity, wind, temperature, and net radiation data.

To calculate MI over the entire country, gridded precipitation, minimum temperature, maximum temperature, solar radiation, and relative humidity were obtained using AWAPer. Gridded PET was calculated using the *Evapotranspiration* package, but since gridded wind speed data is not available for Australia after 2018, we used a modified version of the Penman equation (Valiantzas 2006) that does not require wind speed. The gridded MI was calculated over the pre‐drought (2010–2017) and drought (2018–19) calendar years to visualize drought severity across the continent.

Leaf Area Index (LAI) data were extracted at each site from the MODIS‐based product recently created by the Bureau of Meteorology (unpublished). For the Northern Territory sites, fire frequency estimates from 2000 to 23 were extracted from the Northern Australia Fire Information (NAFI) website over a 1 km radius from each tower location (https://www.ntinfonet.org.au/infonet2/#). At other sites, fire occurrences were reported by the respective Primary Investigators.

### 2.2 Site Classifications and Descriptions

To gain generalizable insights into the effects of the drought on carbon exchange, we grouped sites based on their climate and vegetation structure (see Table S1 for data sources and definitions). Using Principal Components Analysis (PCA), forested sites were grouped into four categories: very dry, dry, seasonally wet, and wet (Table 1). The two grass‐dominated sites (Sturt Plains and Yanco) were grouped together. We opted to create our own groupings rather than use existing classifications to take advantage of the extensive surveys available (Cleverly et al. 2019). This approach also allowed us to avoid known issues with applying standard global vegetation classes in flux studies (Cranko Page et al. 2024).

**TABLE 1:** Site characteristics and PCA groupings (see Figure S2).

	Site (FLUXNET ID)	Dominant species	IGBP/NVIS classes	Reference	Basal area (m^2^/ha)	Biomass (*t*/ha)	SLA (m^2^/kg)	Canopy height (m)	Mean LAI (m^2^/m^2^)	Mean *P* (mm/y)	*P* seasonality
**Grass**	Sturt Plains (AU‐Stp)	C4 grass	GRA/Tussock grasslands	Hutley et al. (2011)	0.8	1.4	11.3	NA	0.5	657	19.2
	Yanco (AU‐Ync)	C3 and C4 grass	GRA/Other cover types	Yee et al. (2015)	No data	No data	No data	NA	1.0	424	0.0
**Very dry**	Alice Springs Mulga (AU‐ASM)	***Acacia aneura*** F.Muell. ex Benth.	SAV/Acacia shrublands	Eamus et al. (2013)	7.0	9.9	2.9	8.1	0.4	390	4.1
	Calperum (AU‐Cpr)	***Eucalyptus socialis*** F.Muell. ex Miq. and ***Eucalyptus dumosa*** A.Cunn. ex J.Oxley	SAV/Mallee woodlands and shrublands	Meyer et al. (2015)	4.3	7.2	3.0	3.9	0.5	267	0.0
	Ti Tree East (AU‐TTE)	***Corymbia opaca*** (D.J.Carr & S.G.M.Carr) K.D.Hill & L.A.S.Johnson and ***A. aneura***	GRA/Hummock grasslands	Tarin et al. (2020)	No data	No data	3.7	4.9	0.2	375	3.9
**Dry**	Cumberland Plain (AU‐Cum)	***Eucalyptus moluccana*** Roxb. and ***Eucalyptus fibrosa*** F.Muell.	EBF/Eucalypt woodlands	Renchon et al. (2018)	19.8	135.5	4.6	26.3	2.8	775	1.5
	Gingin (AU‐Gin)	Mixed banksias	WSA/Other forests and woodlands	Moore et al. (2025)	11.6	52.5	4.0	6.8	1.3	634	−4.2
**Seasonally wet**	Dry River (AU‐Dry)	***Eucalyptus tetrodonta*** F.Muell. and ***Corymbia terminalis*** (F.Muell.) K.D.Hill & L.A.S.Johnson	SAV/Eucalypt open forests	Hutley et al. (2011)	5.3	31.8	4.2	12.7	1.3	961	25.9
	Daly Uncleared (AU‐DaS)	***Eucalyptus tetrodonta*** and ***Corymbia latifolia*** (F.Muell.) K.D.Hill & L.A.S.Johnson	SAV/Eucalypt open forests	Hutley et al. (2011)	7.7	31.0	6.1	19.0	1.7	1232	19.7
	Howard Springs (AU‐How)	***Eucalyptus miniata*** A.Cunn. ex Schauer and ***Eucalyptus. tetrodonta***	WSA/Eucalypt woodlands	Hutley et al. (2022)	9.4	17.6	5.8	20.4	2.4	1848	12.4
	Litchfield (AU‐Lit)	***Eucalyptus miniata*** A.Cunn. ex Schauer and ***Eucalyptus. tetrodonta***	WSA/Eucalypt open forests	Karan et al. (2016)	7.4	40.9	5.8	18.0	2.0	1879	13.0
**Wet**	Tumbarumba (AU‐Tum)	***Eucalyptus delegatensis*** R.T.Baker	EBF/Eucalypt tall open forests	Leuning et al. (2005)	41.2	290.6	5.5	44.4	3.7	1060	−0.5
	Robson Creek (AU‐Rob)	Mixed rainforest trees including ***Flindersia brayleyana*** F.Muell. and ***Litsea leefeana*** (F.Muell.) Merr.	EBF/Rainforests and vine thickets	Bradford et al. (2014)	54.1	505.9	7.7	30.0	5.7	1763	2.4
	Wombat Forest (AU‐Wom)	***Eucalyptus obliqua*** L'Her., ***E. rubida*** Deane & Maiden and ***E. radiata*** Sieber ex DC	EBF/Eucalypt open forests	Hinko‐Najera et al. (2017)	48.5	334.9	6.3	26.4	2.0	888	−0.8

*Note:* All numeric data in this table (excluding that for grasses) are used for PCA. See Table S1 for data sources (which vary between sites) and more detailed definitions.

Abbreviations: IGBP, International Geosphere–Biosphere Programme classification system; LAI, average leaf area index; NVIS, National Vegetation Information System; *P* seasonality, an index that is positive where rainfall is warm‐season dominant and negative where rainfall is cool‐season dominant; *P*, mean annual precipitation from 1990 to 2017; SLA, specific leaf area.

### 2.3 Data Analysis

#### 2.3.1 Drought Severity

To characterize drought severity for each site, we first defined growing years based on NEP seasonality. Specifically, site‐level data were aggregated to the annual timescale beginning in the month of lowest average NEP to avoid splitting growing seasons across years. If the month of lowest NEP occurred before July, the growing year was defined as the same calendar year. If the minimum was during or after July, the growing year was defined as the following calendar year (e.g., 2018 growing year starting in late 2017).

The drought was defined over the 2018–19 growing years for all sites except the seasonally wet sites and Sturt Plains, where the more severe MI deficit was in 2019–20. The pre‐drought comparison period covered 2010 (or start‐of‐record) to 2017 (or 2018 for the seasonally wet sites and Sturt Plains). We defined severe and moderate drought at −30% and −10% of predrought MI respectively.

#### 2.3.2 Quantifying Overall Drought Impacts

For each drought‐affected site, we assessed the overall effect on ecosystem fluxes. We calculated the mean annual ET, ET/*P*, GPP, NEP, and underlying water use efficiency (uWUE = GPPxVPD^0.5^/ET) (Zhou et al. 2014) over the 2 years of drought and compared the values to their pre‐drought averages. Note that the uWUE has been shown to outperform the inherent water use efficiency (Beer et al. 2009) for relating flux tower ET to GPP (Zhou, Yu, et al. 2015). Post‐drought vegetation recovery was evaluated by comparing GPP, ER, and NEP in the first post‐drought year to the most climatically similar pre‐drought year in terms of average MI.

#### 2.3.3 Sensitivity of Annual Fluxes to Water Availability Metrics Across Sites

We investigated overall climate‐productivity relationships to help explain different drought responses across sites. We first compared annual NEP, GPP, and ER with annual rainfall (by growing year). We also tested three additional climate indices as predictors of carbon fluxes, namely the annual MI, the overall water availability deficit (Water Availability Index, WAI), and the water availability deficit in dry months (Monthly Water Deficit Index, MWDI). For the latter two metrics, we first calculated the available water, *X*_*i,j*_, as the difference between *P* and PET in each month (Equation 1):

(1) $$X_{i,j}=P_{i,j}-{\mathrm{PET}}_{i,j}$$

Where i is the monthly timestep and j is the site. To calculate a Water Availability Index (WAI), we summed all values of *X*_*i*_ across growth year *j* (Equation 2).

(2) $${\mathrm{WAI}}_{j}=\sum_{i=1\;}^{12}X_{i}$$

To calculate a Monthly Water Deficit Index (MWDI), we summed all negative values of *X*_*i*_ across growth year *j* (Equation 3).

(3) $${\text{MWDI}}_{j}=\sum_{i=1\;|\;x_{i}<0}^{12}X_{i}$$

Therefore, the WAI represents the overall water availability while the MWDI indicates the exposure to periodic dry conditions. We regressed annual sums of fluxes against the climate indices to determine their influence on site productivity.

#### 2.3.4 Mechanisms Controlling Drought Response

After examining overall drought responses and vegetation‐water relationships, we moved on to a more detailed site‐level analysis to help explain the results. We used 30‐day moving averages of *P*/PET and ET/PET as indicators of water deficit over time and compared them to 30‐day moving averages of NEP. This timescale allowed us to examine sub‐annual effects, such as the use of stored water over dry seasonal periods. This analysis also considered known disturbances at each site.

We next examined leaf‐level drought response mechanisms at the different sites. As per Nelson et al. (2018), we used the divergence in the relative diurnal timing of demand‐adjusted ET (ET/VPD^0.5^) and GPP to indicate photosynthetic limitation by non‐stomatal factors. We calculated and compared the average timing of daily centroids of the two variables for the non‐drought and drought periods. This method is subject to high uncertainty, particularly at drier sites, due to potential changes in the amount and timing of soil evaporation under drought. Our ecosystem‐scale observations are unable to separate these changes from shifts in stomatal behavior.

We attributed additional drought effects on productivity to stomatal closure and/or leaf loss by examining the relative timing of reductions in GPP and LAI. We reason that if GPP declines precede LAI declines, stomatal closure was employed to mitigate water loss before leaf loss. For each site, we calculated monthly *Z*‐scores of MI, GPP, and LAI over 2016–2021. This allowed us to visually compare measurements with different magnitudes and variability. We then took 12‐month moving averages and calculated cross‐correlations (Papoulis 1962) between MI and the two response variables during the transition into drought (2017–2019 calendar years, or 6 months later for seasonally wet sites and Sturt Plains, noting that we did not use growth years here because seasonality is masked in 12‐month moving averages). The cross‐correlation function calculates correlations between two variables over a range of lag times, returning the strongest correlation and the lag time at which it occurs, hence indicating the time taken for MI declines to impact GPP and LAI, respectively.

## 3 Results

### 3.1 Drought Severity at the TERN‐OzFlux Sites

The 2018–19 drought was characterized by high PET and low *P* across most of Australia. The percentage declines in MI relative to pre‐drought averages were largest in central Australia (Figure 1), where MI declined by over 50% across a broad area.

Of the 14 sites included in our analysis, 10 experienced severe drought (MI declined by 30% or more on average) over their respective drought‐affected growing years (Table 2). Two sites experienced relatively small MI declines of 10%–30% (moderate drought) while Robson Creek and Wombat Forest were not subject to drought at the 2‐year timescale.

**TABLE 2:** Change in P and MI between the pre‐drought period and the drought years for the 14 sites.

	Site	DP (%)	DMI	DMI (%)	Growth year starts	Study period (growth years)	Drought period (inclusive)
**Grass**	Sturt Plains	−56	−0.19	***−58***	October	2011–2022	October 2018–September 2020
	Yanco	−47	−0.13	***−54***	January	2013–2021	January 2018–December 2019
**Very dry**	Alice Springs Mulga	−66	−0.13	***−68***	December	2011–2020	December 2017–November 2019
	Calperum	−54	−0.09	***−57***	January	2011–2019	January 2018–December 2019
	Ti Tree East	−66	−0.11	***−67***	December	2013–2021	December 2017–November 2019
**Dry**	Cumberland Plain	−32	−0.18	***−36***	January	2014–2022	January 2018–December 2019
	Gingin	−14	−0.04	*−14*	June	2012–2022	June 2018–May 2020
**Seasonally wet**	Daly Uncleared	−38	−0.24	***−40***	October	2011–2022	October 2018–September 2020
	Dry River	−45	−0.23	***−50***	November	2011–2022	November 2018–October 2020
	Howard Springs	−30	−0.34	***−35***	September	2011–2022	September 2018–August 2020
	Litchfield	−35	−0.39	***−38***	October	2016–2022	October 2018–October 2020
**Wet**	Robson Creek	32	0.30	30	February	2014–2022	February 2018–January 2020
	Tumbarumba	−19	−0.34	*−28*	June	2010–2018	June 2018–May 2020
	Wombat Forest	0.4	−0.02	−3	May	2010–2020	May 2018–April 2020

*Note:* The drought was more severe in the 2019–20 growing years (starting late 2018) for the seasonally wet sites and Sturt Plains, so the drought period and statistics are defined accordingly. Bold italics in the *Δ*MI (%) column indicate severe drought, and italics indicate moderate drought. The growth year begins in the month of lowest NEP for each site, and the study period indicates the growing years for which flux data was available (see Section 2.1 for exclusions related to data issues and disturbance).

### 3.2 Drought Impacts on Annual Fluxes

**FIGURE 2:**
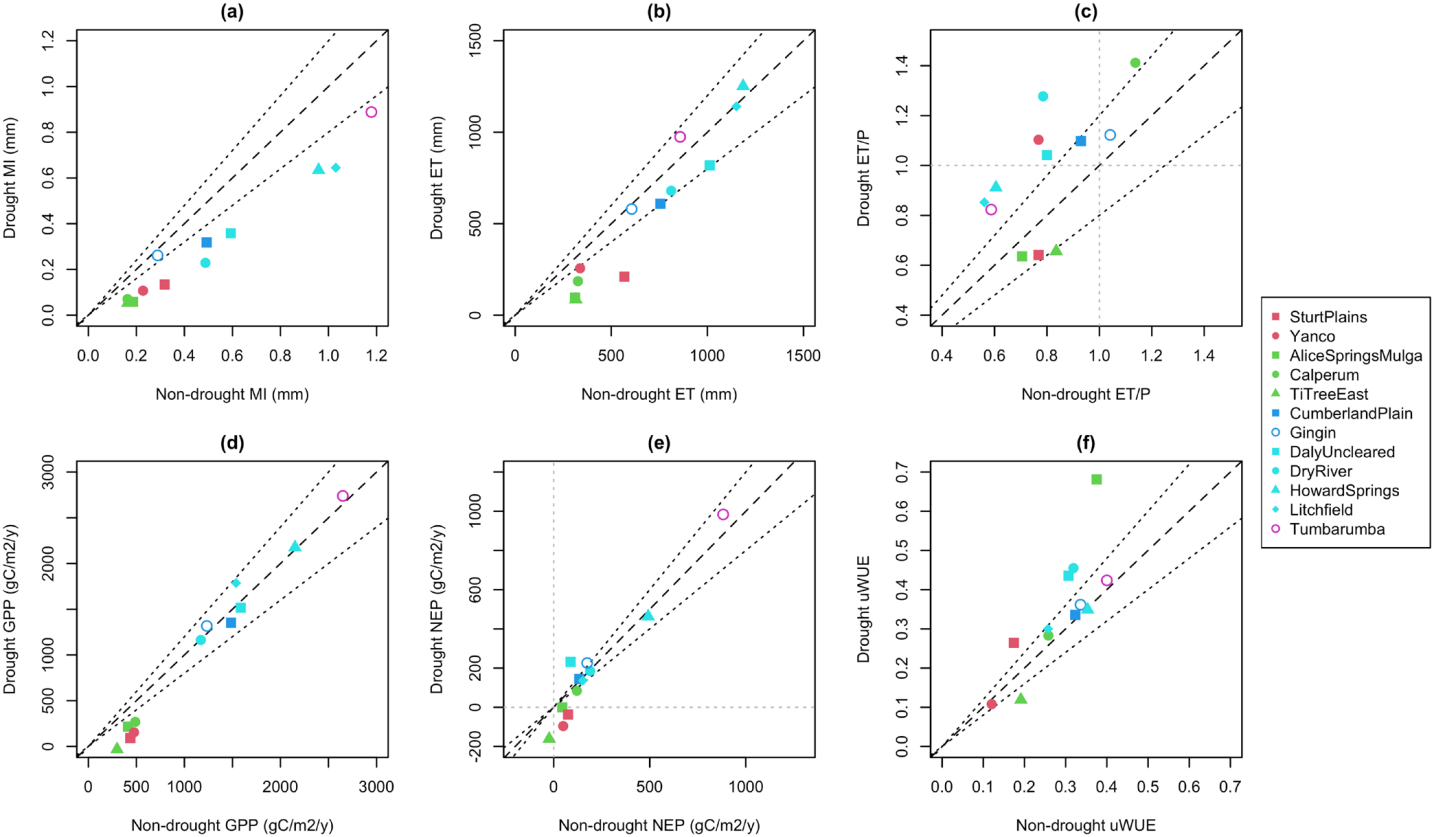
Average values of (a) MI, (b) ET, (c) ET/P, (d) GPP, (e) NEP and (f) *uWUE (GPP* × *VPD* ^*0.5*^/ET) for the drought versus the rest of the post‐2010 TERN‐OzFlux record for each drought‐affected site. Note 2016 at Gingin and 2014 at Calperum are excluded due to fires. Solid symbols indicate severe drought while unfilled symbols indicate moderate drought. The 2019 growing year at Tumbarumba included several months after the fire, so only the 2018 growing year is included. The dashed black lines indicate 1:1 while the dotted black lines show 20% contours. Dotted grey lines indicate (c) ET = *P* and (e) NEP = 0.

The average ET, productivity, and water use efficiency during the drought were compared to pre‐drought averages to quantify drought effects. The results were highly variable across sites, with the largest effects in grass and very dry ecosystems (Figure 2). ET at the sites generally decreased, with reductions of up to 73% (Figure 2b). As expected, most sites consumed a greater fraction of *P* during the drought, with ET/*P* ratios increasing by up to 63% (Figure 2c). However, at some grass and very dry sites, ET was a smaller fraction of *P* during the drought, suggesting hydraulic stress caused a reduced ability to effectively uptake water.

The carbon fluxes indicated remarkable drought resistance at many sites. In particular, the seasonally wet sites maintained or increased their GPP despite large MI reductions (Figure 2d). However, the grass and very dry sites experienced overall declines in GPP, by 45%–100% (average 63%). The GPP averaged across the dry, seasonally wet, and wet drought‐affected sites increased by 2%, indicating no meaningful change in carbon uptake. NEP was generally robust to the drought, with only the grass sites switching from sink to source (Figure 2e). Reduced ER under drought often played a role in maintaining NEP. For example, Daly Uncleared (seasonally wet) saw a large increase in NEP despite no change in GPP, since dry conditions substantially reduced ER. Similarly, a decline in GPP at Calperum (very dry) was compensated by a decline in ER such that average NEP was nearly unchanged by the drought.

The uWUE, a measure of productivity relative to demand‐adjusted water uptake, increased or was maintained during the drought at all sites except for one grass (Yanco) and one very dry (Ti Tree East) site (Figure 2f). While Alice Springs Mulga appears particularly resistant by this measure, the high value during the drought was driven by a reduction in ET that likely reflected reduced soil evaporation rather than more efficient plant water use.

### 3.3 Drought Recovery

**FIGURE 3:**
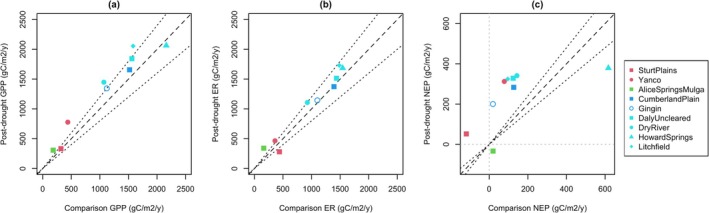
(a) GPP, (b) ER, and (c) NEP in the first post‐drought year (the 2020 growing year except at Sturt Plains and the four seasonally wet sites, for which it is the 2021 growing year) and the pre‐drought year with the most similar MI. Calperum, Ti Tree East and Tumbarumba are excluded due to a lack of data in the first post‐drought year, while Robson Creek and Wombat Forest are excluded because they were not drought‐affected. Dashed black lines indicate 1:1 while dotted black lines show 20% contours. Dotted grey lines show (c) NEP = 0.

To investigate drought resilience, carbon fluxes at the sites in the first post‐drought year were compared against a climatically similar pre‐drought year. Most sites recovered remarkably quickly (Figure 3). GPP in the first post‐drought year was similar or higher than that of the comparison year at all sites (Figure 3a). Only two sites (Alice Springs Mulga and Howard Springs) had lower NEP, driven mostly by higher ER rather than lower GPP (Figure 3c).

### 3.4 Flux‐Climate Relationships

**FIGURE 4:**
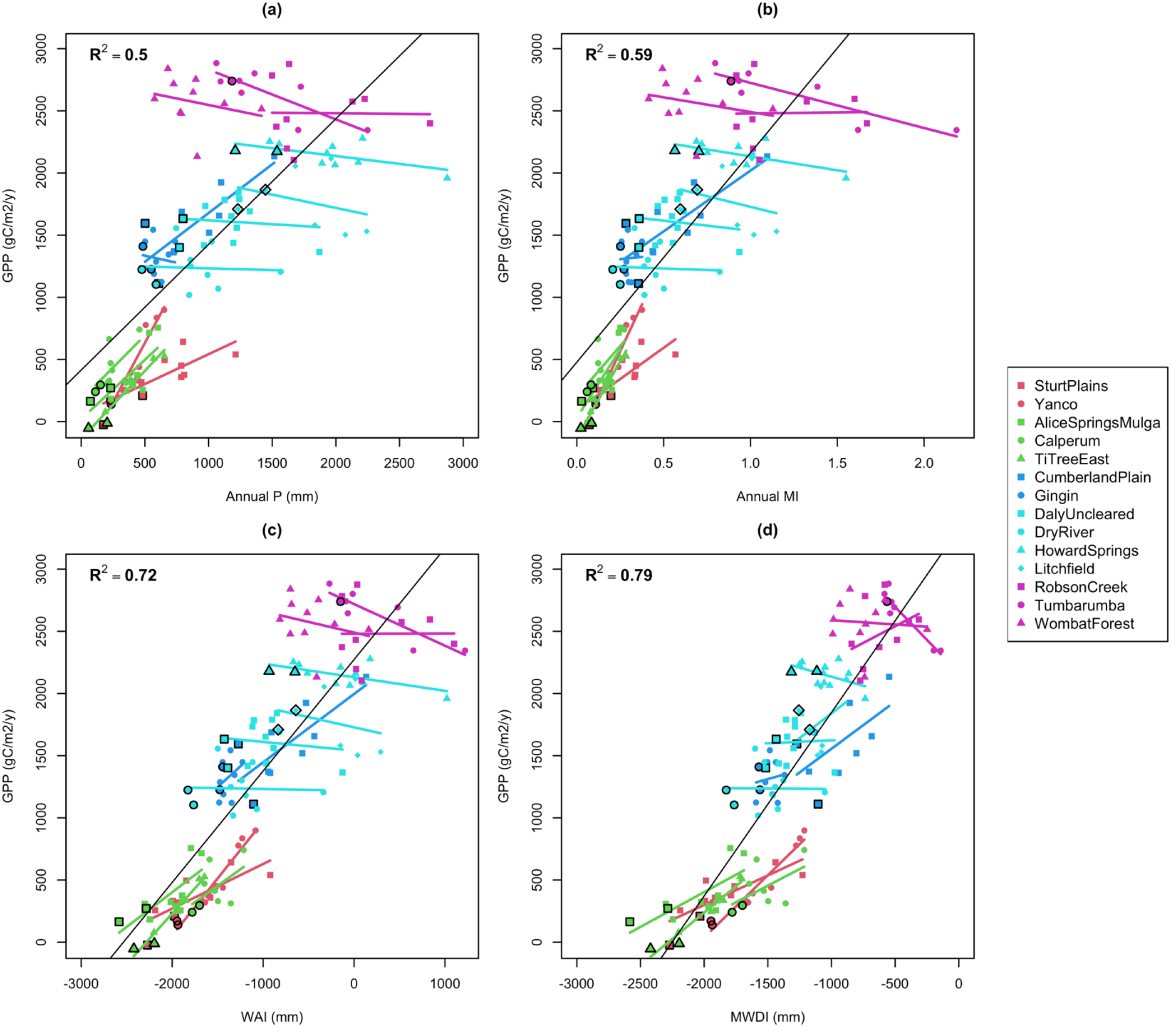
The relationship between annual GPP and (a) annual P, (b) annual MI, (c) WAI, and (d) MWDI with each point representing one growing year. The overall linear fit is shown as a black line, and site‐level fits are shown as coloured lines. The drought years are indicated by black outlines on the points. Note 2016 at Gingin and 2014 at Calperum are excluded due to fires. The 2019 growing year at Tumbarumba included several months after the fire, so only the 2018 growing year is shown.

We examined the overall climate‐productivity relations across the sites to understand whether they could help explain differences in drought vulnerability. *P* (Figure 4a) and MI (Figure 4b) spanned wide ranges at the wet and seasonally wet sites with little influence on productivity. The drier sites responded strongly to *P* and MI and showed more variability in annual GPP, suggesting a “boom‐bust” relationship with water availability. The drought years did not deviate substantially from the overall climate‐GPP relationships.

Of the four climate metrics we tested (annual *P*, annual MI, WAI, and MWDI), MWDI had the strongest explanatory power for predicting GPP overall (Figure 4). MWDI explained 79% of variation in GPP at the annual (growing year) timescale across all 14 sites, as opposed to 50% and 59% for annual *P* and MI, respectively. It also showed a stronger correlation than WAI (72%), driven by a tighter relationship across the wetter sites. The wet sites occur over a large range in annual *P* but did not experience large negative MWDI values during our study period, indicating that their overall exposure to water stress (PET > *P*) is generally low. The seasonally wet sites occur over a similar range in total rainfall but experienced larger negative MWDI values over the study period due to stronger rainfall seasonality, resulting in lower productivity. Overall, our results indicate that ecosystem productivity across space is limited by exposure to sub‐annual periods of water stress.

The MWDI was also a stronger predictor of overall NEP than the other metrics (Table S2, *R* ^2^ = 0.52 for MWDI), further highlighting dry‐period water deficit as a driver of ecosystem function. The correlations between climate and NEP were weaker than GPP due to the confounding influence of ER, which was also dependent on climate and tended to decrease under dry conditions (Figure S3a). Temperature did not have a consistent impact on ER at the annual timescale (Figure S3b). However, ER had a strong positive relationship with GPP both across and within sites, particularly low average‐GPP sites (Figure S3c).

We saw positive site‐level relationships between all four water‐related metrics and GPP for the grass, very dry, and dry sites, indicating that water availability explains year‐to‐year productivity. However, in the seasonally wet and wet sites, no water availability metric we tested explained within‐site temporal variability in productivity. Therefore, our results imply that water stress can explain GPP across space, but not across time except at relatively dry sites.

### 3.5 Temporal Dependence of NEP on Climate

**FIGURE 5:**
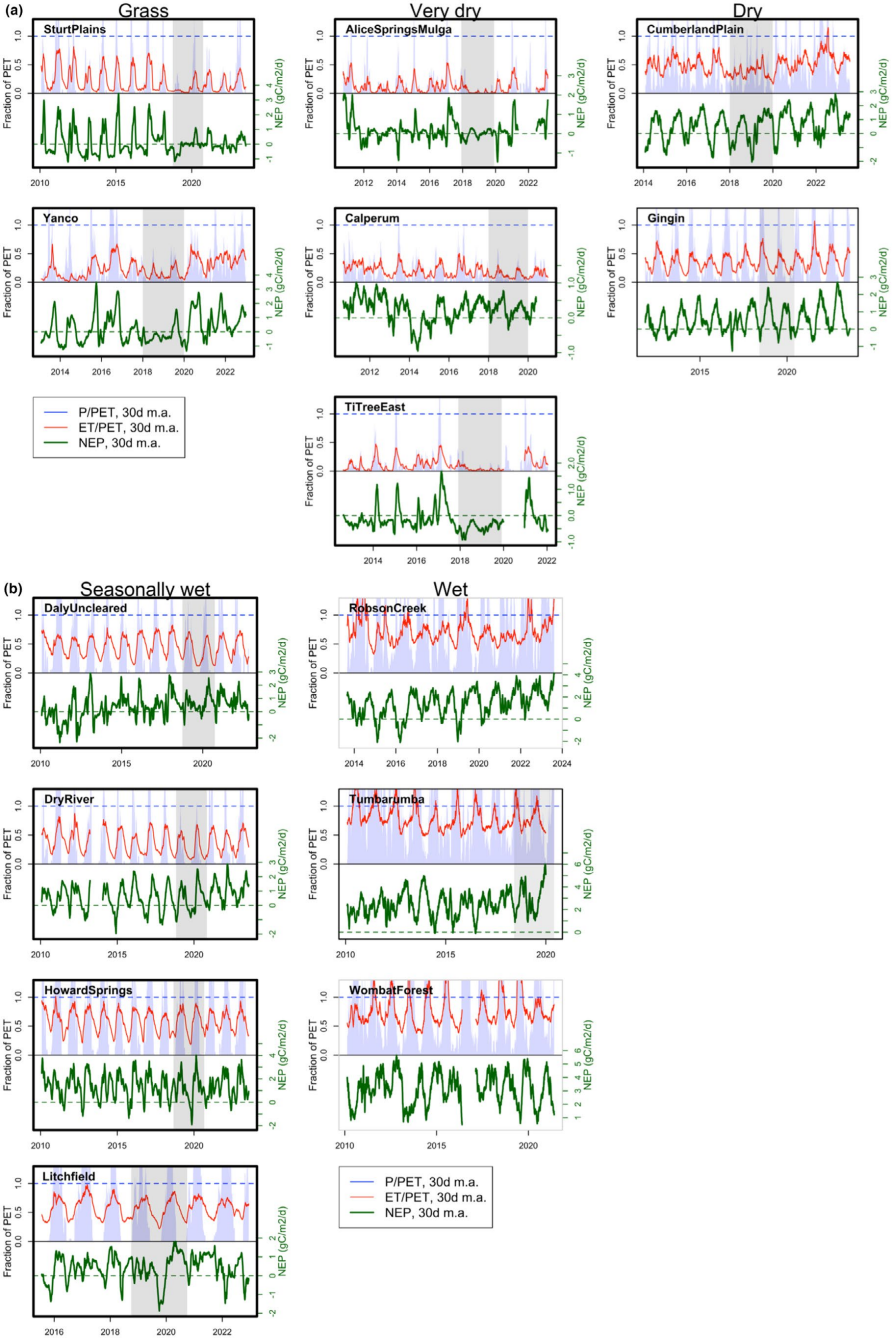
(a) 30‐day moving averages of MI (P/PET) and ET/PET for each grass, very dry, and dry site, together with NEP in the lower part of each panel. NEP above zero indicates a carbon sink while values below zero indicate a source. The blue dashed line indicates *P* = PET (relative to blue shading) or ET = PET (relative to red line). Bold black borders indicate that a site experienced severe drought while thin black borders indicate moderate drought. Grey shading highlights the drought period. Fires occurred at Calperum in 2014 and Gingin in 2016. (b) 30‐day moving averages of MI (P/PET) and ET/PET for each seasonally wet and wet site, together with NEP in the lower part of each panel. NEP above zero indicates a carbon sink while values below zero indicate a source. The blue dashed line indicates *P* = PET (relative to blue shading) or ET = PET (relative to red line). Bold black borders indicate that a site experienced severe drought while thin black borders indicate moderate drought and grey borders indicate no drought. Grey shading highlights the drought period. Howard Springs, Litchfield, Daly Uncleared, Dry River, and Sturt Plains experienced around 20, 15, five, three, and one fire(s) respectively between 2000 and 2023 according to NAFI estimates, with a high severity fire noted at Litchfield in 2019.

To develop deeper insight into the controls on water use and productivity during the extreme drought at each site, we examined time series of MI, ET/PET, and NEP (Figure 5, noting variables are 30‐day moving averages). MI is a measure of the climatic water deficit, while ET/PET indicates the water consumption at the land surface relative to evaporative demand. By tracking how ET/PET (red line) follows MI (*P*/PET, blue shading), we can deduce how strongly ET is controlled by climate within the same 30‐day period. If MI is higher than ET/PET, some rainfall is going into site runoff (surface or subsurface) or soil water storage. If ET/PET is higher than MI, plants are accessing stored soil water to maintain ET over a period of low *P*. The time course of NEP (green line) and whether it tracks ET/PET reflects the degree of coupling between carbon and water fluxes.

At the most water‐limited sites (grass and very dry, Figure 5), ET/PET closely tracked MI, indicating that most *P* was consumed quickly as ET except in brief high‐rainfall periods. NEP responded strongly to incoming water, so both ET/PET and NEP declined as MI fell during the drought. Calperum differed somewhat from the other very dry sites: the pattern of ET/PET was similar, but NEP was less responsive to water limitation. This could relate to access to deep soil moisture, noting that ET/PET remained above zero at Calperum even when *P*/PET approached zero.

The dry ecosystems differed from grass and very dry sites in their notable access to stored *P* (shown by ET/PET exceeding MI in dry periods, Figure 5). This stored water access buffered low rainfall periods so that NEP was not strongly affected by reduced soil water availability during the drought. Interestingly, even during very wet periods (*P* > > PET), PET was rarely met by ET. This finding suggests that water uptake during wet periods was limited by the vegetation's ability to transport water, indicating the long‐term emergence of ecosystems that balance wet‐period and dry‐period productivity. Soil water stores enabled by reduced wet‐period water use appeared to enable productivity over dry periods, a mechanism that probably contributed to drought resistance in 2018–19. At Cumberland Plain, the low‐rainfall 2018 growing year had a lower NEP peak than any other year (Figure 5, also see Figure S4 for NEP plotted against day‐of‐year, with years overlain to aid comparison), but NEP recovered remarkably in 2019, which was still relatively dry. This behavior has been attributed to pre‐drought mistletoe infestation of eucalypts that increased their water stress but was cleared by branch drop in 2018 (Griebel et al. 2022). At Gingin, measurements reflect a fuel reduction burn in 2016 that reduced NEP. Post‐fire recovery likely drove high NEP in subsequent years (including during the drought).

The seasonally wet sites showed a strong seasonal pattern in water use (Figure 5b). MI consistently exceeded ET/PET during the wet season (indicating infiltration or runoff), but ET/PET was higher than MI during the dry season (indicating stored water use). These sites clearly depended on stored wet‐season rainfall to maintain ET over the dry season. Despite high water availability during summer, PET was rarely met by ET, suggesting that the ecosystems' capacity to uptake water during wet periods was regulated by long‐term adjustments in response to dry periods (similar to the dry sites). NEP responded to ET/PET at the seasonal timescale, but the relationship was unclear at the interannual timescale. At Daly Uncleared, Dry River, and Litchfield, peak NEP was relatively low in 2019, which was a dry year. However, all three sites had much higher growing season NEP in 2020 despite similarly dry conditions.

NEP in the three wet ecosystems showed little evidence of water limitation (Figure 5b). While seasonal periods of low *P*/PET did occur, access to stored water meant that ET rarely dropped below half of PET at the 30‐day timescale.

Overall, our time series analysis indicates that access to stored soil water during times of atmospheric water deficit is a key factor that defines drought resistance of the different ecosystem types. Even during the severe, widespread 2018–19 drought, the dry, seasonally wet, and wet sites maintained ET, while the grass and very dry sites were forced to reduce their ET and hence their productivity. Access to groundwater sourced from non‐local *P* can be indicated by ET > *P* over long time periods; we did not see clear evidence of this at any of the sites.

### 3.6 Physiological Drought Effects

Drought can affect the productivity of an ecosystem via several mechanisms, including leaf loss, stomatal closure, and non‐stomatal limitation of photosynthesis through changes in internal leaf processes (e.g., reduced electron transport efficiency or mesophyll conductance). Increased non‐stomatal limitation of photosynthesis was most evident at one very dry site (Alice Springs Mulga). The centroid of ET/VPD^0.5^ shifted earlier in the day (on average) during autumn (March—May) and spring (September—November), indicating earlier stomatal closure to avoid water loss (Figure S5), but this shift was not seen in the diurnal GPP timing (Figure S6). Greater decoupling between ET/VPD^0.5^ and GPP was also evident for Ti Tree East in spring and Sturt Plains in summer. Non‐stomatal limitations to photosynthesis may have contributed to higher drought sensitivity at these sites relative to another very dry site, Calperum. However, our results may overstate the extent of non‐stomatal limitation at sites with substantial soil evaporation, as noted in Section 2.3.4. Overall, we did not find evidence for a dominant role of non‐stomatal factors in limiting GPP under drought.

**FIGURE 6:**
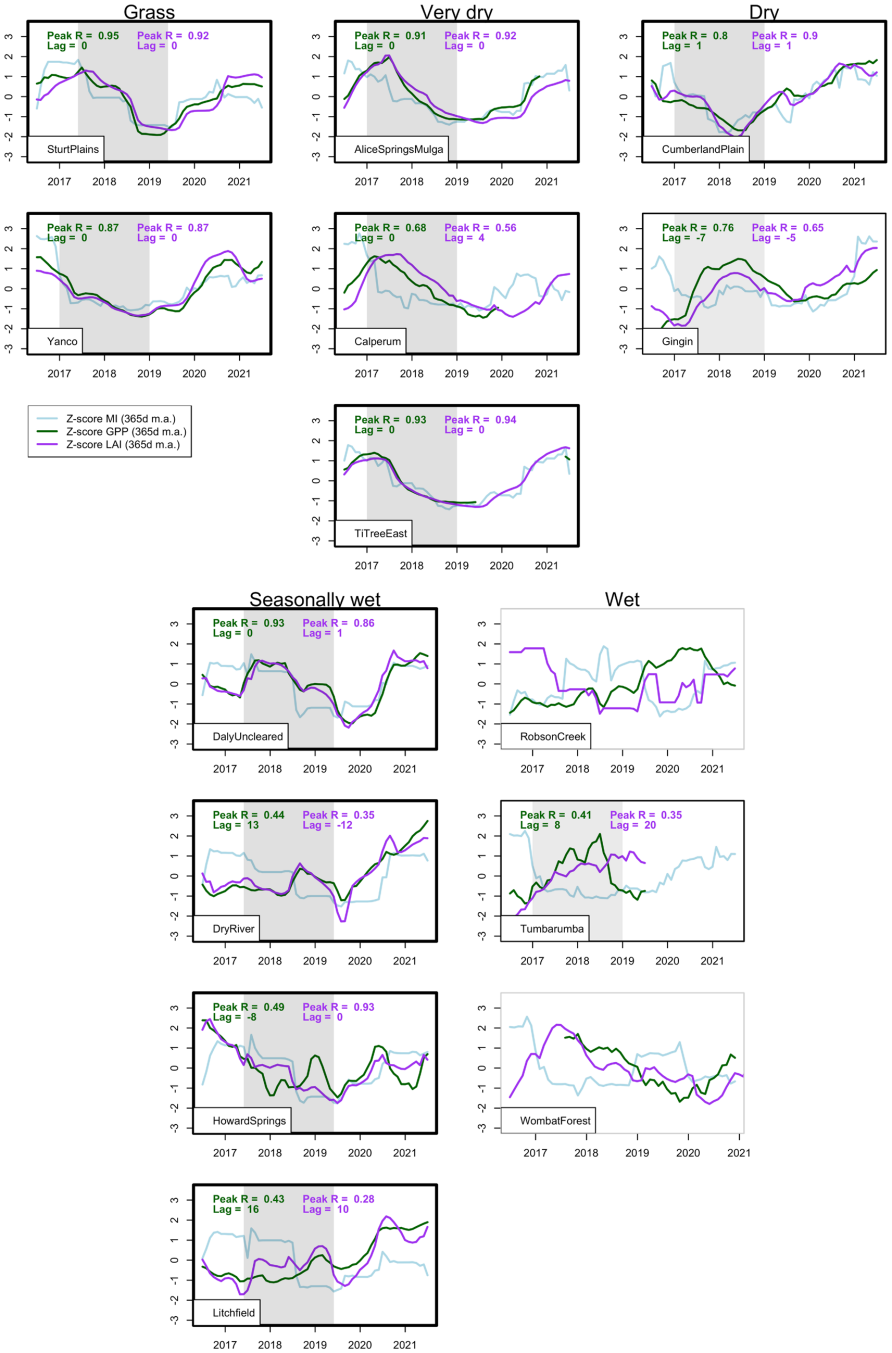
Standardised 12‐month moving averages of MI, GPP, and LAI over the transition into peak drought (01/01/2019 to 31/12/2018 for most sites, shifted 6 months later for Sturt Plains and the seasonally wet sites). Note that the moving averages are centred on the plots, so the point on 01/01/2018 is the average of data from the second half of 2017 and the first half of 2018 (for example). The text indicates peak correlations and lags between 12‐month moving averages of *Z*‐scores for MI and GPP (green)/LAI (purple) during transition into drought. The timeframe covered by the cross‐correlations is indicated by grey shading. Thick black borders around the plots indicate sites that were subject to severe drought while thin black borders indicate moderate *drought* and grey borders indicate no drought.

In the absence of non‐stomatal drought effects on photosynthesis, the importance of leaf loss versus stomatal closure can be inferred through the relative timing of LAI and GPP changes (Figure 6). The peak correlations between MI declines and GPP/LAI declines during the transition into drought were strong (mostly > 0.7) at the grass, very dry, and dry sites, as well as Daly Uncleared. The lags suggest that leaf loss occurred quickly with declining MI at the grass sites, Ti Tree East and Alice Springs Mulga (very dry), Cumberland Plain (dry), and Daly Uncleared (seasonally wet), with zero or 1‐month lags between MI and GPP/LAI shifts. At Calperum (very dry), the correlation between MI and GPP peaked 3 months before the correlation between MI and LAI, suggesting an initial stomatal response to drying with some delay before leaves senesced.

At the seasonally wet sites other than Daly Uncleared, the correlations between MI and GPP/LAI were weak (mostly < 0.7) and the lags were often not physiologically meaningful (e.g., response variables appearing to shift earlier than the driving climate variable), possibly because the response to drought was confounded by frequent fires, seasonal light limitation, and complex tree‐grass growth dynamics. At the wet site (Tumbarumba), the LAI increased early in the drought and was maintained until the fire. However, GPP began to decline through 2019. This suggests that the vegetation may have become water stressed well into the drought and employed stomatal closure but not leaf loss.

## 4 Discussion and Conclusions

### 4.1 Drought Response

Our continental‐scale analysis of ecosystem response to the severe 2018–19 drought showed substantial reductions in productivity in the five driest (grass and very dry) ecosystems only, where GPP declined by 65% on average (or 1.0 g C m^−2^ year^−1^ per mm rainfall reduction). Three of these five ecosystems switched from being carbon sinks to sources (Figure 2). At three sites, a smaller fraction of local rainfall evaporated during the drought than in pre‐drought years, suggesting detrimental drought effects on vegetation health that impeded water uptake (Plaut et al. 2013) as opposed to adaptive downregulation of ET. However, in wetter ecosystems (those classified as dry, seasonally wet, or wet), vegetation showed remarkable resistance to drought with no overall reduction in GPP or NEP. The contrasting responses confirm that reduced rainfall was most impactful at the driest sites (Biederman et al. 2016; Huxman et al. 2004; Scott et al. 2015).

By examining the control of *P* and PET on ET and NEP (Figure 5), we identified that the primary factor enabling surprising drought resistance at the dry and seasonally wet sites was access to stored water during dry periods (consistent with the findings of Rungee et al. (2019) in the western US). At these intermediately water‐limited sites, soil moisture was not fully depleted and ET continued even over a severe, multi‐year drought. During wet periods (*P* >> PET), ET generally stayed well below PET at the monthly timescale, indicating long‐term emergence of ecosystems where wet‐period ET is modulated by dry‐period water stress. A potential mechanism is transport limitation of ET during wet periods due to lower overall vegetation density than would be sustained in the absence of dry periods. This reduction in wet‐season water use could then contribute to larger soil moisture stores that sustain productivity over dry periods. Patterns of water use where ET variability is “dampened” relative to rainfall variability have been demonstrated before in savanna trees (Eamus et al. 2000; O'Grady et al. 1999), and may be especially common in Australia's highly variable climate (Nicholls et al. 1997). Ecosystems exposed to high climate variability are known to be more robust to changing climate averages (Nathan et al. 2019). Therefore, exposure to seasonal water deficits could explain why flux sensitivity to drought in Australia is considered low relative to parts of Europe, North America, and Asia (Schwalm et al. 2010).

Given that a large fraction of the Australian land area is characterised by grassland or arid woodlands, drought vulnerability in these ecosystems has major implications for carbon storage in the landscape. The literature on drought resistance in dry regions is mixed. Some studies find that semi‐arid and sub‐humid areas are most sensitive because vegetation is more drought‐adapted in arid ecosystems (Yang et al. 2016; Zhang et al. 2021), while we found the strongest GPP decreases at the driest sites. The discrepancy likely relates to drought severity—very dry ecosystems may be well adapted to maintain productivity under mild to moderate drought but downregulate or even experience mortality under severe drought. Productivity in grasslands is consistently found to be vulnerable to soil water deficits (Deng et al. 2023; Hoek van Dijke et al. 2023; Hoover and Rogers 2016). We generally noted small or even positive drought impacts on GPP and NEP in wetter ecosystems, likely associated with lower cloud cover and higher radiation, which aligns with previous studies in other parts of the world (Miller et al. 2023; Sousa et al. 2020). ER and GPP were positively correlated at all sites, indicating that carbon loss in years of low productivity was buffered by corresponding low respiration (Figure S3c). We did not find consistent evidence of higher respiration in warmer years (Figure S3b), which contrasts with the findings of von Buttlar et al. (2018) at shorter timescales.

We noted that the seasonally wet sites (all tropical savannas) showed relatively low NEP during the first year of drought, but not during the second year (Figure 5). Australian savannas experience regular fire (Russell‐Smith and Yates 2007), and it is possible that high productivity in the years leading up to the 2018–19 drought led to above‐average burn severity in 2019. Supporting this idea, a high severity fire was recorded at Litchfield in 2019 (Luck et al. 2023). Reduced fire risk due to lower biomass the following year, together with vegetation regrowth, may have driven higher carbon uptake in 2020 despite continuing drought. Peak growing season NEP at Daly Uncleared and Dry River was low in some particularly wet years (e.g., 2011), suggesting a role of light limitation. Previous studies at Howard Springs (the wettest of the four sites, Table 1) show that the majority of GPP is concentrated in the wet season and that growth during this time is limited by light (Kanniah et al. 2013; Moore et al. 2016, 2018; Whitley et al. 2011).

The drought‐affected ecosystems showed remarkable resilience. In the first post‐drought year, GPP matched or exceeded GPP during a climatically similar pre‐drought year at all sites (Figure 3). This contrasts with previous findings of negative drought legacy effects on GPP at the annual timescale in Europe and the United States (Anderegg et al. 2015; Yu et al. 2022). Negative legacy effects are generally expected due to declining non‐structural carbohydrate stores, reduced carbon allocation to leaves, or xylem damage induced by drought (Kannenberg et al. 2020). However, these factors may be compensated for by soil nutrient accumulation during drought‐induced periods of low uptake (Shen et al. 2016; Stephens et al. 2023) or upregulation of photosynthesis to facilitate canopy repair (Kannenberg et al. 2019).

### 4.2 Flux‐Climate Relationships

We found that average GPP across different Australian ecosystems was explained by sub‐annual periods of water stress, as opposed to average water availability. Annual GPP combined across all sites correlated strongly with MWDI, a metric that measures the atmospheric water deficit in months where *P* < PET (Figure 4d). Relationships of *P*, MI, and WAI with GPP were weaker at the wetter end of the climate spectrum. The strong explanatory power of MWDI suggests that a key determinant of overall productivity across different parts of Australia is the frequency and severity of water deficit. It seems that high productivity sites are only able to establish and persist in locations that experience limited periodic water deficit.

None of the water availability metrics explained year‐to‐year productivity in individual seasonally wet or wet ecosystems (Figure 4) and NEP did not respond clearly to MI or ET/PET except at seasonal timescales (Figure 5b). It therefore seems that, while overall ecosystem potential for productivity depends on exposure to periodic water stress, year‐to‐year GPP variability in wetter ecosystems is controlled by other factors (e.g., incoming radiation and disturbance). This finding is consistent with previous work indicating light limitation at wet eucalypt (van Gorsel et al. 2013) and savanna (Kanniah et al. 2013; Moore et al. 2018) sites. We suggest that future work could focus on further untangling the drivers of interannual GPP variability for the individual wet and seasonally wet ecosystems.

In contrast, interannual GPP variability at grass and very dry sites depended strongly on water availability (Figure 4). These drier sites tended to be less productive relative to overall water availability, probably due to phenological constraints and/or higher proportional water loss via soil evaporation (Scott et al. 2021), and had less access to subsurface stores in dry periods. It follows that the wetter ecosystems (which have emerged in response to the dry end of their climatic range and are co‐limited by resources other than water) are less likely to experience reduced productivity than the drier ecosystems (where productivity is strongly moisture‐limited), even during severe drought. An exception is when drought leads to other disturbances in wetter ecosystems, as occurred in 2019 with the fire at Tumbarumba. Disturbance events influenced carbon uptake at several of our study sites, highlighting the need to account for individual site histories when interpreting drought responses in flux data.

### 4.3 Drought Resistance Strategies

The timing of changes in different variables can give clues to the mechanisms underlying drought response. Six sites (two grass, two very dry, one dry, and one seasonally wet) showed declining LAI within a month of reduced GPP (Figure 6), suggesting that drought stress was only minimally mitigated by stomatal closure and leaf thinning/senescence occurred rapidly. In contrast, at Calperum (the southernmost very dry site), stomatal closure may have been employed to mitigate water stress, and leaf loss only occurred around 3 months later. Calperum has the lowest average rainfall of the three very dry sites but also the lowest PET and the deepest soils/roots, which may contribute to lower drought stress. Additionally, it is possible that hotter sites (further north) benefit from a more rapid leaf loss response because fallen leaves could slow soil evaporation. The temporal relationships between climate and LAI/GPP were relatively unclear at three of the four seasonally wet sites. However, in line with expectations from field studies (not published), the timing of GPP changes generally matched the timing of LAI changes (Figure 6), indicating leaf loss (as opposed to stomatal regulation) was the vegetation's primary water‐saving strategy. At Tumbarumba (wet), GPP began declining in early 2019, suggesting drought stress and stomatal closure, but LAI was maintained until wildfire impacted the site on 31/12/2019.

### 4.4 Drought Mortality

Previous work has highlighted mortality in eastern Australian eucalypt forests during the 2018–19 drought (De Kauwe et al. 2020; Losso et al. 2022; Nolan et al. 2021), whereas we found high drought resistance at the drought‐affected eucalypt sites (Calperum, Cumberland Plain, the seasonally wet sites, and Tumbarumba). While there was some surplus tree mortality recorded at Cumberland Plain (Griebel et al. 2022), it is unclear how much this dieback contributed to the small decrease in 2018 NEP relative to other years. Pre‐drought mistletoe infestation complicated dynamics at Cumberland Plain, and the drought‐related processes that cleared the mistletoe ultimately seemed to benefit the eucalypts. Observed drought mortality has often been mediated by terrain and root zone conditions; for example, tree death may be more likely in upslope areas that do not receive water subsidies or on north‐facing slopes with higher incoming radiation (Keppel et al. 2023; Mattos et al. 2023). These cases are unlikely to be captured by eddy covariance towers, which are best located in flat areas (Aubinet et al. 2012; Moore and Griebel 2024). This siting constraint presents a fundamental limitation to upscaling from flux towers, since vegetation communities potentially at highest risk of drought stress and mortality are unlikely to be captured. Given the absence of flux towers in eucalypt forests that did experience substantial drought mortality, future work should combine relevant field observations from 2018 to 19 with remote sensing and modeling approaches to further investigate the implications for water and carbon fluxes.

### 4.5 Implications for Model Development

A key aim of our analysis was to provide information for developing, parameterizing, and validating process‐based vegetation models during periods of drought. The overall climate‐productivity relationships we quantified could be used to benchmark modeled plant‐water relations. We also identified key drivers of variation in drought response across ecosystem types, which could inform model development. For example, our results point to subsurface water storage as an important factor in drought resistance and overall climate‐productivity dynamics, but current models are known to have deficiencies around processes like root water uptake (van Oorschot et al. 2021) and preferential flow (Gharari et al. 2019). Assuming one global value for soil depth is common and may limit model performance, for example, the standard soil depth in LPJ‐GUESS is currently 1.5 m (Zhou et al. 2024). We did not find evidence for substantial access to non‐locally sourced groundwater at the study sites, yet this is a known factor in other ecosystems (Doody et al. 2017; Mu et al. 2021) and is lacking in many current models (Zeng et al. 2018).

Our analysis of plant drought response mechanisms can inform model development and parameterization. We showed that vegetation at most sites (excluding wet sites) tended to drop leaves rapidly under drought stress, although one very dry eucalypt site (Calperum) employed stomatal closure first. Connecting phenology and allocation strategies to water availability (as opposed to only temperature, as in many current models) could lead to performance improvements in many parts of the world, including most of Australia. We only found evidence of increased non‐stomatal photosynthetic limitation under drought at three of the driest sites but note limitations in the method we used, particularly at sites with substantial soil evaporation. Previous work using leaf‐level measurements, which would likely be more reliable than eddy covariance data for detecting changes in physiological function, has pointed to the high importance of non‐stomatal photosynthetic responses to water stress (Egea et al. 2011). Therefore, it remains an important consideration for model improvement (Zhou, Wang, et al. 2015; Zhou et al. 2013).

Overall, our results highlight the diversity in drought responses across different ecosystems and underscore the need for landscape models that can capture this variation. Given projections of future drying in many regions, managing ecosystem health will require an understanding of which environments are most at risk. By examining responses to extreme climatic events, we can refine process representation in models to inform ecological management in a rapidly changing environment.

## Author Contributions

**C. Stephens:** conceptualization, formal analysis, investigation, methodology, project administration, validation, visualization, writing – original draft, writing – review and editing. **B. Medlyn:** conceptualization, investigation, methodology, resources, supervision, writing – review and editing. **L. Williams:** methodology, validation, writing – review and editing. **J. Knauer:** writing – review and editing. **A. Inbar:** writing – review and editing. **E. Pendall:** data curation, writing – review and editing. **S. K. Arndt:** data curation, writing – review and editing. **J. Beringer:** data curation, writing – review and editing. **C. M. Ewenz:** data curation, writing – review and editing. **N. Hinko‐Najera:** data curation, writing – review and editing. **L. B. Hutley:** data curation, writing – review and editing. **P. Isaac:** data curation, methodology. **M. Liddell:** data curation, writing – review and editing. **W. Meyer:** data curation, writing – review and editing. **C. E. Moore:** data curation, writing – review and editing. **J. Cranko Page:** methodology, writing – review and editing. **R. Silberstein:** data curation, writing – review and editing. **W. Woodgate:** data curation, writing – review and editing.

## Conflicts of Interest

The authors declare no conflicts of interest.

## Supporting information


Data S1.

## Data Availability

The data and code that support the findings of this study are openly available in Zenodo at http://doi.org/10.5281/zenodo.15833540. Flux data was obtained from the Terrestrial Ecosystem Research Network at https://doi.org/10.25901/2jwh‐qq50 (Alice Springs Mulga), https://doi.org/10.25901/cvb1‐d129 (Calperum), https://doi.org/10.25901/hw22‐a072 (Cumberland Plain), https://doi.org/10.25901/wf22‐1n09 (Daly Uncleared), https://doi.org/10.25901/bm7t‐f932 (Dry River), https://doi.org/10.25901/shfz‐mn52 (Gingin), https://doi.org/10.25901/kvx6‐m534 (Howard Sprin), https://doi.org/10.25901/68tc‐d142 (Litchfield), https://doi.org/10.25901/zdwj‐xv49 (Robson Creek), https://doi.org/10.25901/4qm0‐8893 (Sturt Plains), https://doi.org/10.25901/tt9q‐pz64 (Ti Tree East), https://doi.org/10.25901/fxpp‐pb92 (Tumbarumba), https://doi.org/10.25901/32jz‐2j52 (Wombat State), and https://doi.org/10.25901/352y‐2j72 (Yanco). Ecoplots and TERN core hectare data were obtained from the TERN discovery portal at https://portal.tern.org.au/. Specific leaf area was estimated from AusTraits data available from Zenodo at https://doi.org/10.5281/zenodo.3568417 and downloaded via the “traitecoevo/austraits” R package. Climate data were obtained from the Australian Water Availability Project via the BoM repository at http://www.bom.gov.au/web03/ncc/www/awap/rainfall/totals/daily/grid/0.05/history/nat/ for rainfall using the AWAPer R package. For Howard Springs and Litchfield, alternative rainfall data was obtained from the Bureau of Meteorology Climate Data Online Portal at http://www.bom.gov.au/climate/data/ (gauges Howard Springs Nature Park and Walker Creek). LAI data were obtained from the Bureau of Meteorology MODIS‐based product, which was developed to better capture the Australian landscape. The gridded product is currently under review but can be accessed from Australia's National Computing Infrastructure with permission from the Bureau of Meteorology (BoM).
