# Supplementary material for: The Response and Recovery of Carbon and Water Fluxes in Australian Ecosystems Exposed to Severe Drought
Source: Glob Chang Biol. 2025 Jul 25;31(7):e70361. doi: 10.1111/gcb.70361 (PMC12291013; doi:10.1111/gcb.70361)
Supplement: Supplementary file 1 — Data S1. [file GCB-31-e70361-s001.docx]

# Supplementary

Table S1 Data sources for site classification through PCA

| **Variable** | **Primary data source** | **Exceptions** |
| --- | --- | --- |
| Basal area | Accessed from the TERN Data Discovery Portal (<https://portal.tern.org.au/>). Values are calculated based on diameter measurements from the TERN core 1-hectare survey data.  In the case of multiple surveys, we took the survey with the highest basal area (to avoid drought or fire-affected measurements). | Diameter data for Gingin provided directly by site PI (pers. comm).  The value for Wombat was taken from Bennett (2016).  Because surveys were not available for Ti Tree East, we used values from the nearest site (Alice Springs Mulga) as estimates for the PCA.  No data was available for Yanco. |
| Biomass | Ecoplots data accessed from the TERN Data Discovery Portal (<https://portal.tern.org.au/>). Values are calculated based on diameter measurements from the TERN core 1-hectare survey data and site-specific allometric equations. Data provided individually for each site (Arndt & Hinko-Najera, 2022; Boer & Pendall, 2022; Bradford et al., 2024; Keith et al., 2022; Meyer, 2022; Silberstein, 2024).  We used values from the Biomass Plot Library (Joint Remote Sensing Research Program, 2021) for Alice Springs Mulga, Dry River, Daly Uncleared, Howard Springs, Litchfield, and Sturt Plains due to missing or suspect data in Ecoplots.  In the case of multiple surveys, we took the survey with the highest biomass (to avoid drought or fire-affected measurements). | Diameter data for Gingin provided directly by site PI (pers. comm), processed using the allometric equation for *Banksia grandis* in Hingston et al. (1990).  Because surveys were not available for Ti Tree East, we used values from the nearest site (Alice Springs Mulga) as estimates.  No data was available for Yanco. |
| Specific leaf area (SLA) | Mean value for each dominant species taken from AusTraits (Falster et al., 2021), then averaged across up to four dominant species for each site. | For Wombat, SLA for *Eucalyptus obliqua* sourced from literature (Pritzkow, Szota, et al., 2020; Pritzkow, Williamson, et al., 2020).  No data was available for the dominant species at Yanco. |
| Canopy height | 95^th^ percentile of individual tree heights from core 1-ha survey data, accessed from the TERN Data Discovery Portal (<https://portal.tern.org.au/>). | Taken from Cleverly et al. (2016) for Ti Tree East, Karan et al. (2016) for Litchfield, and pers. comm for Gingin. |
| LAI | Bureau of Meteorology LAI product (manuscript currently under review) based on MODIS data (Myneni et al., 2015) but processed specifically for Australia, averaged across 2010-2017 (pre-drought study period). | Value for Wombat Forest taken from Griebel et al. (2015), MODIS severely overestimates. |
| Precipitation | AWAP data downloaded using AWAPer R package (Peterson et al., 2020) and extracted at each site location over 1990-2017. | Bureau of Meteorology daily rainfall gauge data used for Howard Springs (14149) and Litchfield (14279) due to low rainfall bias in AWAP data. Where gauge data was reported as accumulated over multiple days, total was averaged over relevant days. Gaps infilled with AWAP data. |
| Precipitation seasonality | Ratio of warm season (October through March) to cool season (April through September) rainfall, minus the ratio of cool season to warm season rainfall over 1950-2017. Positive values imply summer-dominated rainfall while negative values imply winter-dominated rainfall. |  |

*Warra and Whroo were excluded from the analysis due to data gaps in 2018/19, while Great Western Woodlands was excluded because of unexplained increases in GPP following an instrument change in 2019.

# Table S1 References

Arndt, S., & Hinko-Najera, N. (2022). *Wombat Stringybark Eucalypt Stem Diameter, Height and Aboveground Woody Biomass Data* [Dataset]. TERN. https://portal.tern.org.au/metadata/TERN/a17c90ad-3f6a-4993-87e0-4135b78969b1

Bennett, A. (2016). *What the flux? High eddy covariance NEP in a dry sclerophyll eucalypt forest is validated using inventory and growth models* [Masters]. University of Melbourne.

Boer, M., & Pendall, E. (2022). *Cumberland Plain Stem Diameter, Height and Aboveground Woody Biomass Data* (Version 1.0) [Dataset]. TERN. https://portal.tern.org.au/metadata/TERN/35bdcb46-17f6-455d-9a98-b540b98e9aea

Bradford, M., McKeown, A., Ford, A., & Liddell, M. (2024). *Robson Creek Rainforest Diameter, Height and Aboveground Woody Biomass Data* (Version 1.0) [Dataset]. TERN. https://portal.tern.org.au/metadata/TERN/5a864ec1-f780-4104-a242-7ff00e1f4962

Cleverly, J., Eamus, D., Van Gorsel, E., Chen, C., Rumman, R., Luo, Q., Coupe, N. R., Li, L., Kljun, N., Faux, R., Yu, Q., & Huete, A. (2016). Productivity and evapotranspiration of two contrasting semiarid ecosystems following the 2011 global carbon land sink anomaly. *Agricultural and Forest Meteorology*, *220*, 151–159. https://doi.org/10.1016/j.agrformet.2016.01.086

Falster, D., Gallagher, R., Wenk, E. H., Wright, I. J., Indiarto, D., Andrew, S. C., Baxter, C., Lawson, J., Allen, S., Fuchs, A., Monro, A., Kar, F., Adams, M. A., Ahrens, C. W., Alfonzetti, M., Angevin, T., Apgaua, D. M. G., Arndt, S., Atkin, O. K., … Ziemińska, K. (2021). AusTraits, a curated plant trait database for the Australian flora. *Scientific Data*, *8*(1), 254. https://doi.org/10.1038/s41597-021-01006-6

Griebel, A., Bennett, L. T., Culvenor, D. S., Newnham, G. J., & Arndt, S. K. (2015). Reliability and limitations of a novel terrestrial laser scanner for daily monitoring of forest canopy dynamics. *Remote Sensing of Environment*, *166*, 205–213. https://doi.org/10.1016/j.rse.2015.06.014

Hingston, F. J., Galbraith, J. H., & Jones, M. S. (1990). *Dimensional Data for Trees at Several Sites in Northern Jarrah Eucalyptus Marginata Forest* (No. 11; User Series). CSIRO Division of Foerstry and Forest Products.

Joint Remote Sensing Research Program. (2021). *Biomass Plot Library—National collation of stem inventory data and biomass estimation, Australian field sites.* (Version 1.0) [Dataset]. TERN. https://portal.tern.org.au/metadata/TERN/fc4a7249-ebb2-4ada-8e06-b552bfb297a3

Karan, M., Liddell, M., Prober, S. M., Arndt, S., Beringer, J., Boer, M., Cleverly, J., Eamus, D., Grace, P., Van Gorsel, E., Hero, J.-M., Hutley, L., Macfarlane, C., Metcalfe, D., Meyer, W., Pendall, E., Sebastian, A., & Wardlaw, T. (2016). The Australian SuperSite Network: A continental, long-term terrestrial ecosystem observatory. *Science of The Total Environment*, *568*, 1263–1274. https://doi.org/10.1016/j.scitotenv.2016.05.170

Keith, H., Stol, J., & Woodgate, W. (2022). *Tumbarumba Wet Eucalypt Stem Diameter, Height and Aboveground Woody Biomass Data* [Dataset]. TERN. https://portal.tern.org.au/metadata/TERN/d6f199ed-aea6-4c18-90e6-89b1c6d4dc81

Meyer, W. (2022). *Calperum Mallee Stem Diameter, Height and Aboveground Woody Biomass Data* [Dataset]. TERN. https://portal.tern.org.au/metadata/TERN/c8d054b2-9dfc-4328-8251-f0fd9276083d

Myneni, R., Knyazikhin, Y., & Park, T. (2015). *MOD15A2H MODIS Leaf Area Index/FPAR 8-Day L4 Global 500m SIN Grid V006* [Dataset]. https://doi.org/10.5067/MODIS/MOD15A2H.006

Peterson, T. J., Wasko, C., Saft, M., & Peel, M. C. (2020). AWAPer: An R package for area weighted catchment daily meteorological data anywhere within Australia. *Hydrological Processes*, *34*(5), 1301–1306. https://doi.org/10.1002/hyp.13637

Pritzkow, C., Szota, C., Williamson, V. G., & Arndt, S. K. (2020). Phenotypic Plasticity of Drought Tolerance Traits in a Widespread Eucalypt (Eucalyptus obliqua). *Forests*, *11*(12). https://doi.org/10.3390/f11121371

Pritzkow, C., Williamson, V., Szota, C., Trouvé, R., & Arndt, S. K. (2020). Phenotypic plasticity and genetic adaptation of functional traits influences intra-specific variation in hydraulic efficiency and safety. *Tree Physiology*, *40*(2), 215–229. https://doi.org/10.1093/treephys/tpz121

Silberstein, R. (2024). *Gingin Banksia Woodlands Stem Diameter, Height, Basal Area and Aboveground Woody Biomass Data* [Dataset]. TERN. https://portal.tern.org.au/metadata/TERN/019eaa3c-d6a6-40c8-92d4-f79c4efa4f0b

Table S2 Relationship (R^2^ values) between ecosystem growth variables (GPP, ER, and NEP) and climate indicators (P, MI, WAI, and MWDI) at the annual timescale (growing years).

|  | **GPP** | **ER** | **NEP** |
| --- | --- | --- | --- |
| **Annual P** | 0.50 | 0.62 | 0.14 |
| **Annual MI** | 0.59 | 0.66 | 0.28 |
| **WAI** | 0.72 | **0.72** | 0.37 |
| **MWDI** | **0.79** | 0.71 | **0.52** |


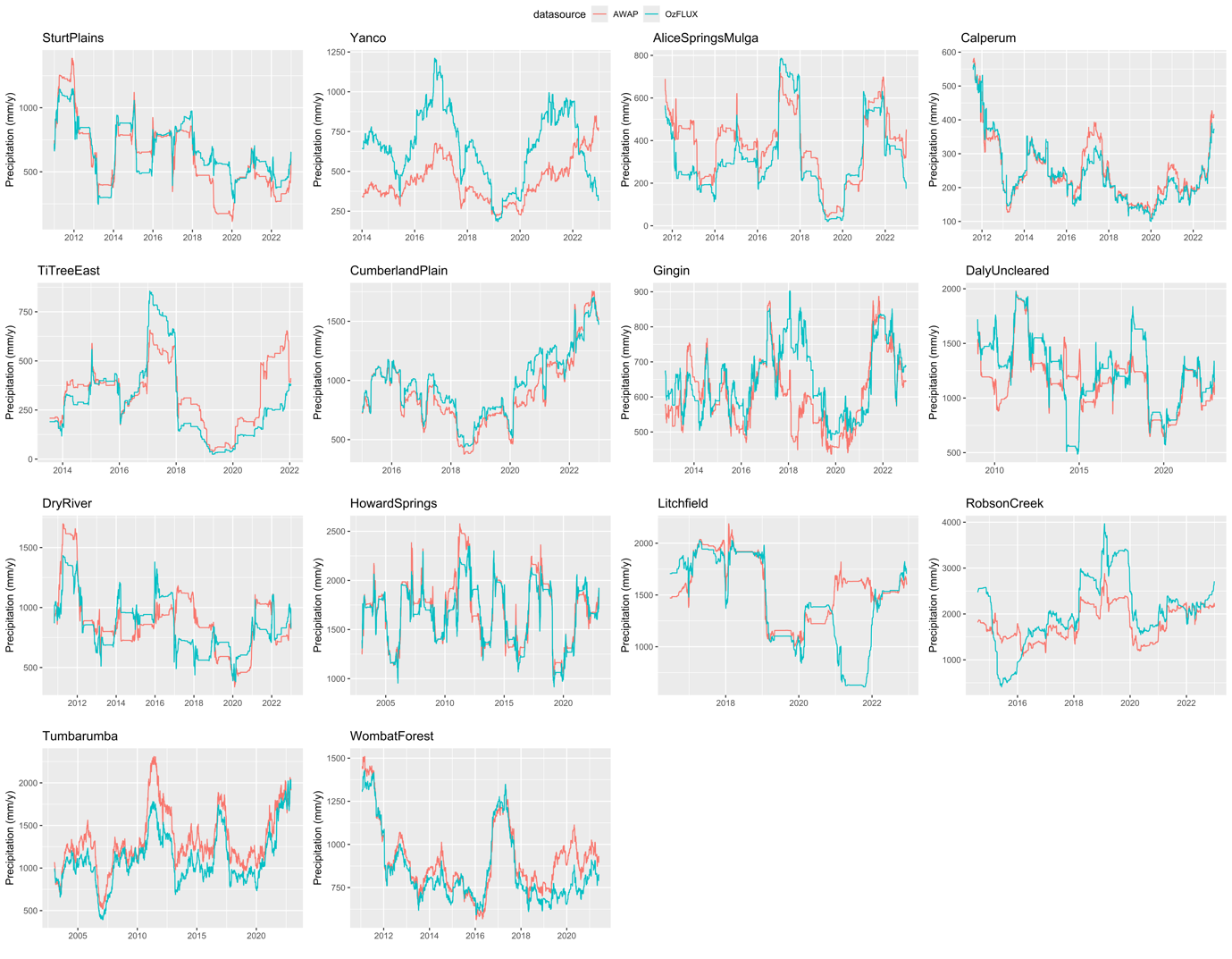


Figure S1 Comparison between 365-day moving sum of rainfall recorded at TERN-OzFlux rain gauges (blue) versus values extracted from the AWAP gridded product (red).


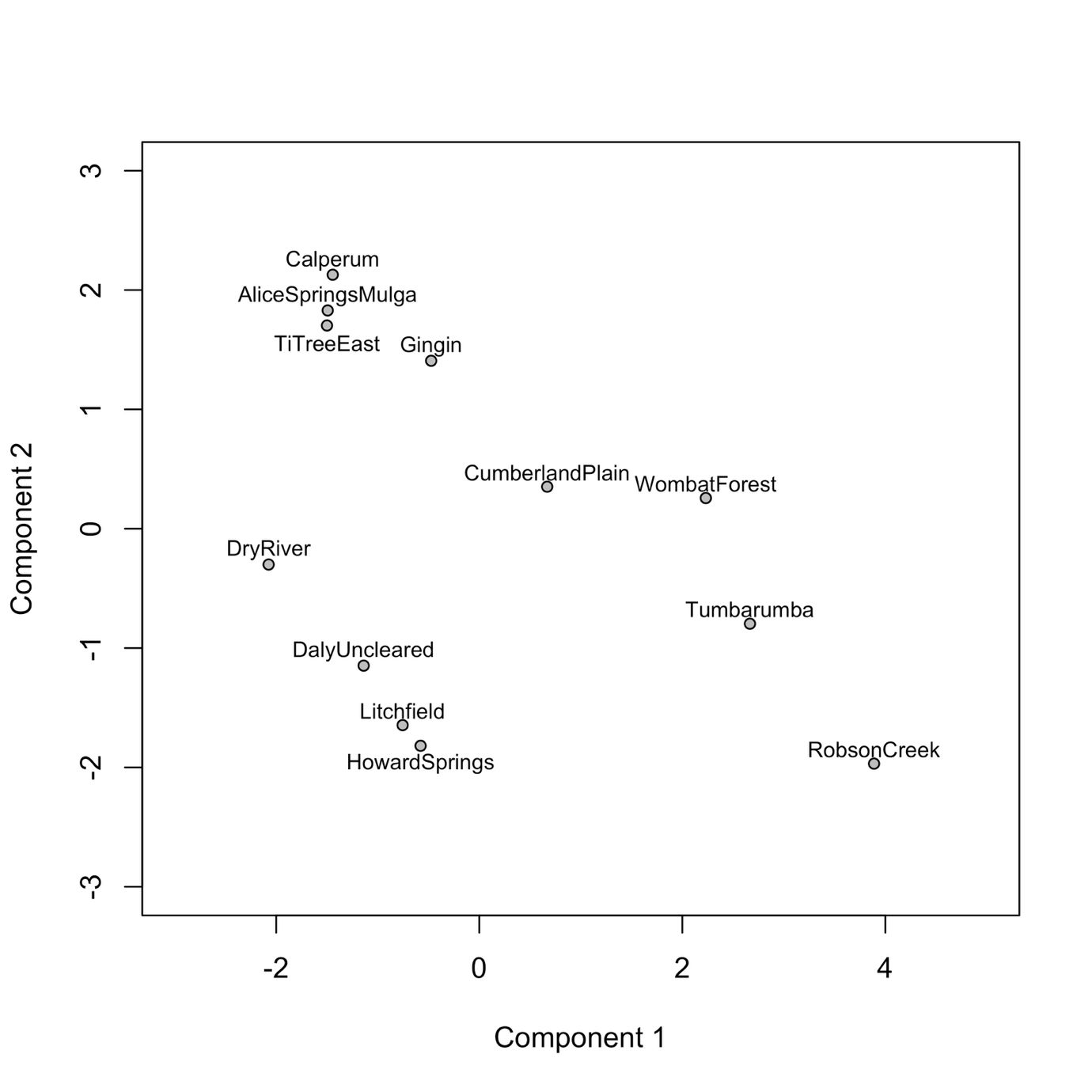


Figure S2 Results of PCA to group ecosystem types. Component 1 explained 93% of the variance between sites while Component 2 explained a further 7%. We grouped the sites ensuring at least two within each class. Gingin falls (marginally) closest to Ti Tree East, but we elected to group it with Cumberland Plain. This choice is considered appropriate in light of our understanding of water relations at the respective sites.


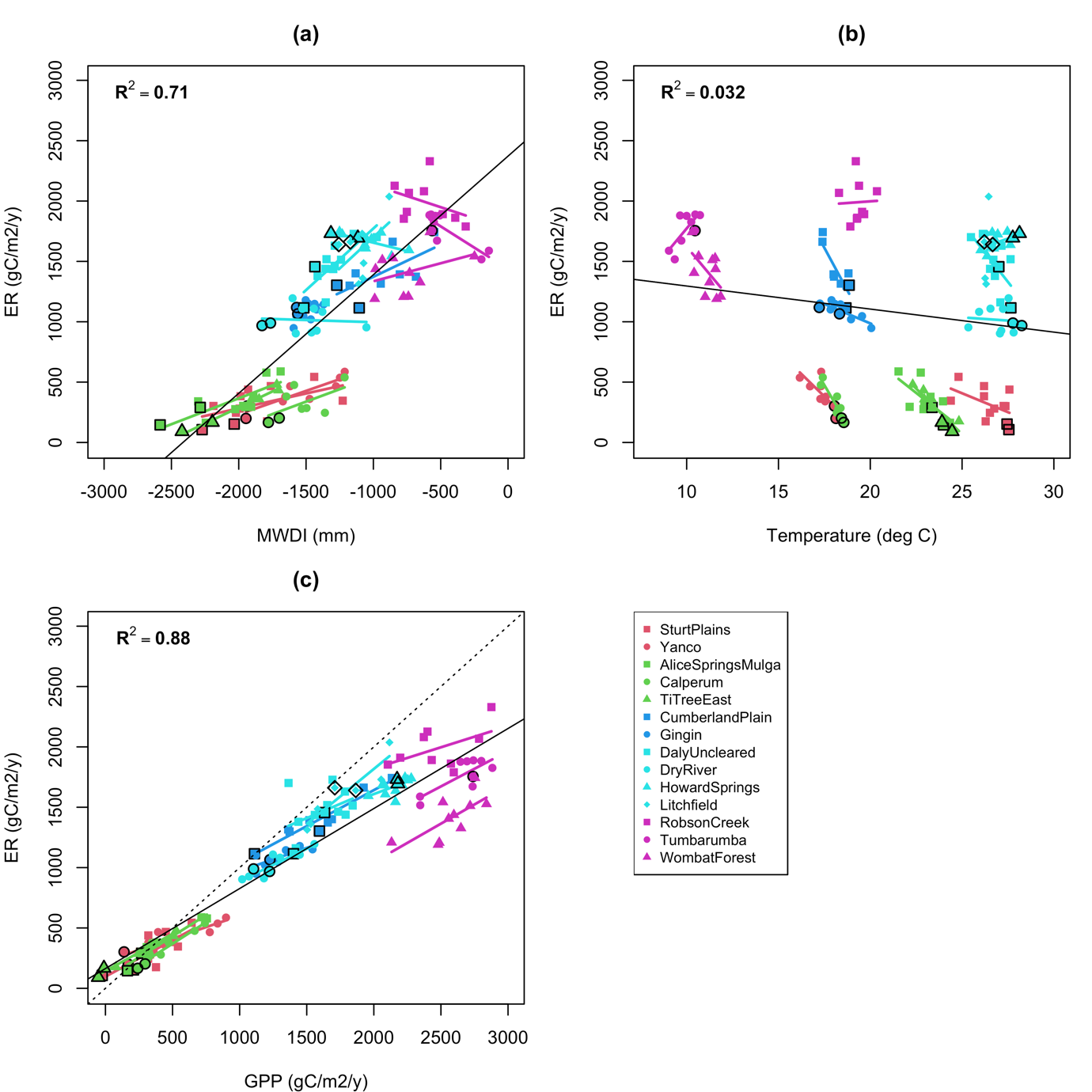


Figure S3 Relationship between ER and (a) MWDI, (b) average air temperature, and (c) GPP across the 14 TERN-OzFlux sites, calculated at the annual (growing year) timescale. Black outlines on the points indicate drought years. The solid black line on each plot indicates the linear fit to the data while the dotted line indicates 1:1. Note 2016 at Gingin and 2014 at Calperum are excluded due to fires. ER at Wombat Forest is known to be underestimated due to the terrain surrounding the site (pers. comm).


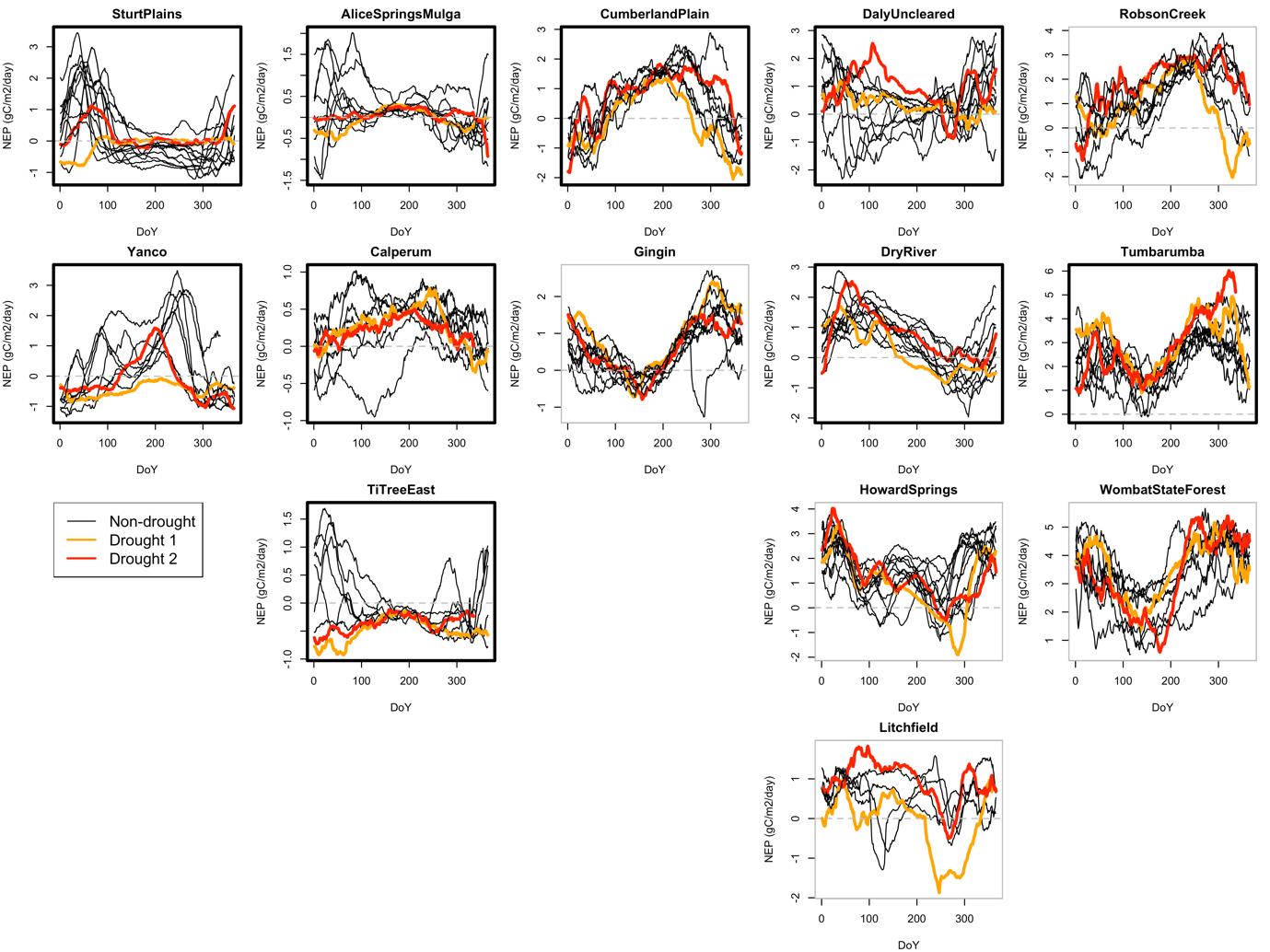


Figure S4 30-day moving average of NEP plotted by day-of-year across all years within the study period for each site. The drought years, generally 2018 and 2019 but 2019 and 2020 for Sturt Plains and the seasonally wet sites, are shown in orange and red.


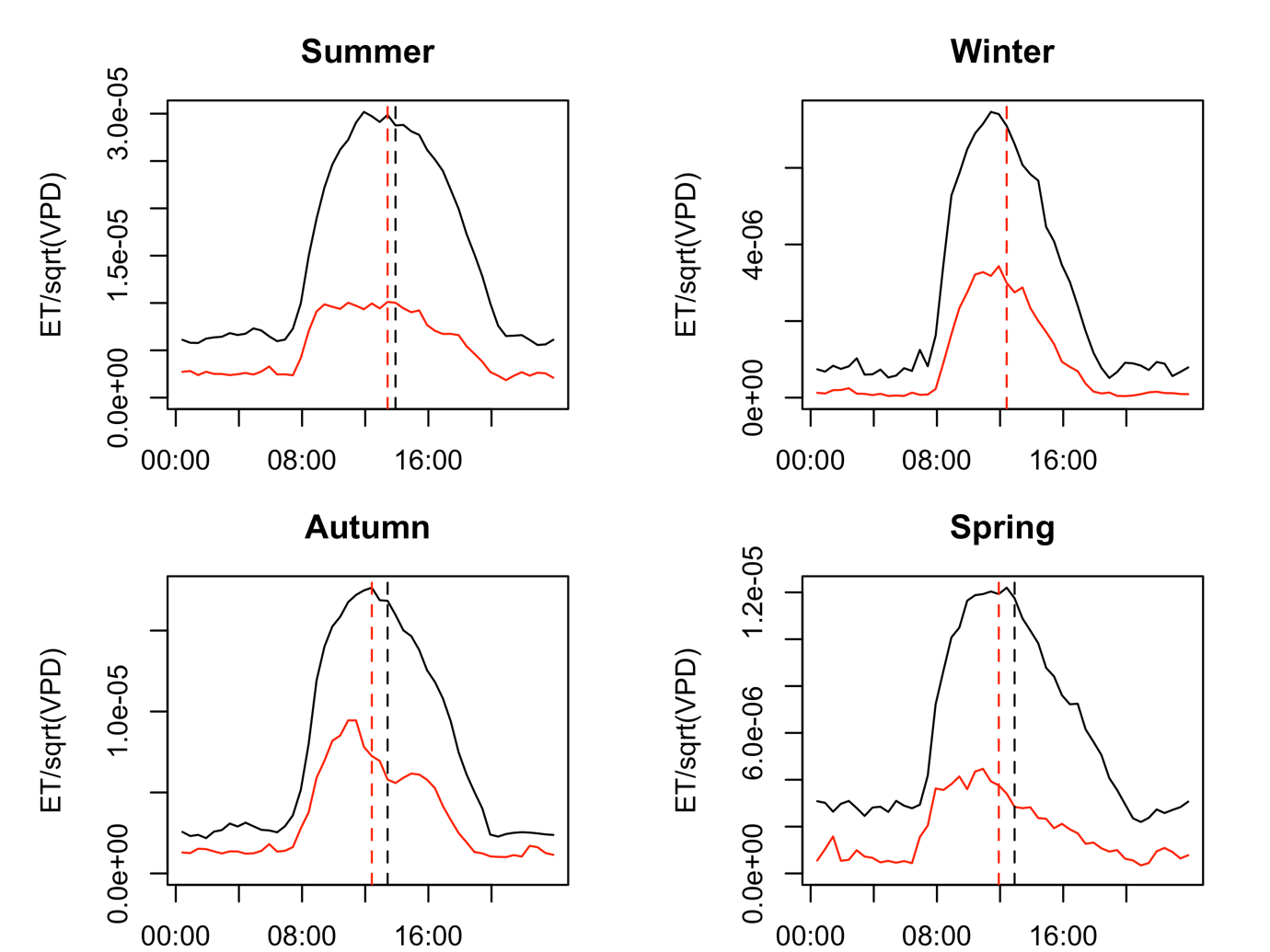


Figure S5 Average diurnal patterns of demand-adjusted water use by season at Alice Springs Mulga during drought (red) and non-drought (black) periods. The centroid is shown as a dashed line. Note that the drought and non-drought timing coincides in winter so only one line is visible. Summer is December-February, autumn is March-May, winter is June-August, and spring is September-November.


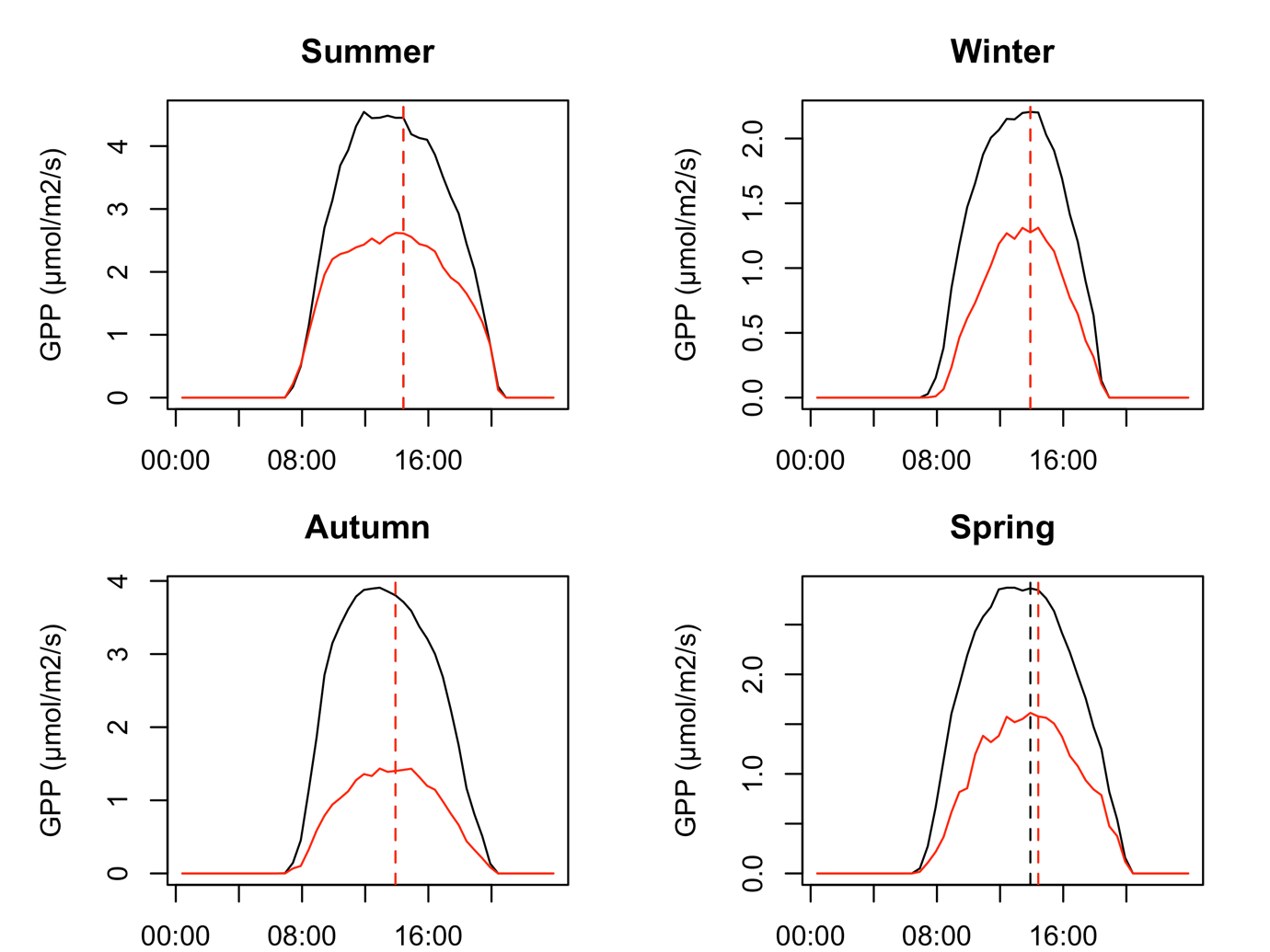


Figure S6 Average diurnal patterns of GPP by season at Alice Springs Mulga during drought (red) and non-drought (black) periods. The centroid is shown as a dashed line. Note that the drought and non-drought timing coincides in summer, autumn, and winter so only one line is visible. Summer is December-February, autumn is March-May, winter is June-August, and spring is September-November.
